# Temporal dynamics of soil microbial C and N cycles with GHG fluxes in the transition from tropical peatland forest to oil palm plantation

**DOI:** 10.1128/aem.01986-24

**Published:** 2024-12-23

**Authors:** Frazer Midot, Kian Mau Goh, Kok Jun Liew, Sharon Yu Ling Lau, Mikk Espenberg, Ülo Mander, Lulie Melling

**Affiliations:** 1 Sarawak Tropical Peat Research Institute, Kota Samarahan, Sarawak, Malaysia; 2 Faculty of Science, Universiti Teknologi Malaysia 538525 , Skudai, Johor, Malaysia; 3 Institute of Ecology and Earth Sciences, University of Tartu 117222 , Tartu, Estonia; University of Delaware, Lewes, Delaware, USA

**Keywords:** metagenomics, methane, nitrous oxide, carbon dioxide, tropical peat swamp forest

## Abstract

Tropical peatlands significantly influence local and global carbon and nitrogen cycles, yet they face growing pressure from anthropogenic activities. Land use changes, such as peatland forests conversion to oil palm plantations, affect the soil microbiome and greenhouse gas (GHG) emissions. However, the temporal dynamics of microbial community changes and their role as GHG indicators are not well understood. This study examines the dynamics of peat chemistry, soil microbial communities, and GHG emissions from 2016 to 2020 in a logged-over secondary peat swamp forest in Sarawak, Malaysia, which transitioned to an oil palm plantation. This study focuses on changes in genetic composition governing plant litter degradation, methane (CH_4_), and nitrous oxide (N_2_O) fluxes. Soil CO_2_ emission increased (doubling from approximately 200 mg C m^−2^ h^−1^), while CH_4_ emissions decreased (from 200 µg C m^−2^ h^−1^ to slightly negative) following land use changes. The N_2_O emissions in the oil palm plantation reached approximately 1,510 µg N m^−2^ h^−1^, significantly higher than previous land uses. The CH_4_ fluxes were driven by groundwater table, humification levels, and C:N ratio, with *Methanomicrobia* populations dominating methanogenesis and *Methylocystis* as the main CH_4_ oxidizer. The N_2_O fluxes correlated with groundwater table, total nitrogen, and C:N ratio with dominant *nirK*-type denitrifiers (13-fold *nir* to *nosZ*) and a minor role by nitrification (a threefold increase in *amoA*) in the plantation. *Proteobacteria* and *Acidobacteria* encoding incomplete denitrification genes potentially impact N_2_O emissions. These findings highlighted complex interactions between microbial communities and environmental factors influencing GHG fluxes in altered tropical peatland ecosystems.

**IMPORTANCE:**

Tropical peatlands are carbon-rich environments that release significant amounts of greenhouse gases when drained or disturbed. This study assesses the impact of land use change on a secondary tropical peat swamp forest site converted into an oil palm plantation. The transformation lowered groundwater levels and changed soil properties. Consequently, the oil palm plantation site released higher carbon dioxide and nitrous oxide compared to previous land uses. As microbial communities play crucial roles in carbon and nitrogen cycles, this study identified environmental factors associated with microbial diversity, including genes and specific microbial groups related to nitrous oxide and methane emissions. Understanding the factors driving microbial composition shifts and greenhouse gas emissions in tropical peatlands provides baseline information to potentially mitigate environmental consequences of land use change, leading to a broader impact on climate change mitigation efforts and proper land management practices.

## INTRODUCTION

Peatlands, which cover approximately 3% of the Earth’s land mass—around 423 million hectares—extend from the tropics to the Arctic (1). Peatlands store a third of the world’s soil carbon and a tenth of its soil nitrogen despite covering a relatively small terrestrial area (2). Tropical peatlands, estimated at 44–170 million hectares, are critical carbon and nitrogen reservoirs, containing approximately 20% of global soil carbon and 6% of global soil nitrogen (2, 3). These peatlands are primarily located in coastal areas or inland basins of Southeast Asia, Central Africa, and South America (4–7). Conserving tropical peatlands can contribute to climate change mitigation by safeguarding their natural carbon storage capabilities (8). However, socioeconomic pressures have led to significant changes in land use in these ecosystems, including logging, pulpwood planting, agriculture, and construction (9). Such disturbances have led to significant emissions of greenhouse gases (GHGs) such as carbon dioxide (CO_2_), methane (CH_4_), and nitrous oxide (N_2_O), which vary depending on the extent and type of disturbance (10).

The conversion of tropical peatlands to agricultural land has profound environmental consequences (11). Factors, such as temperature, groundwater level, peat humification, and soil nutrient levels, could influence GHG fluxes, but their impact may vary, particularly with agricultural development that alters groundwater levels to meet crop needs (12–14). Changes in aboveground vegetation can affect soil microclimate, peat formation, and physicochemical properties (15). Drainage, commonly used to lower groundwater levels in tropical peatlands, has been linked to increased soil CO_2_ levels and shifts in microbial communities (16, 17). Given the large carbon stock of tropical peatlands, GHG emissions driven by microbial communities in response to land use changes are concerning (14, 18, 19). Identifying and understanding variations in GHG emissions associated with different land uses is crucial, especially considering the high global warming potential of CH_4_ and N_2_O (20, 21). Soil microorganisms, particularly prokaryotes, drive the production and consumption of GHGs through carbon and nitrogen compound transformation (22, 23). Pressure from land use changes can shift microbial communities, affecting peat formation, carbon turnover, and nutrient mineralization that support ecosystem sustainability (24).

Therefore, a comprehensive understanding of microbial communities and their functional properties is key to infer ecosystem responses to land use change, including microbial contributions to GHG emissions (25). Methane, a potent GHG, can be produced anaerobically by methanogens through methanogenesis from CO_2_, methanol, methylamines, methyl sulfides, and acetate (26). Conversely, CH_4_ is converted back to CO_2_ by reverse methanogenesis and through CH_4_ oxidation by aerobic methanotrophic bacteria and anaerobic methanotrophic archaea or bacteria (26). Methanogens and methanotrophs also play roles in the nitrogen cycle. Methanogenesis coupled with nitrogen fixation to facilitate the input of nitrogen compounds into anoxic soils (27). Anaerobic methanotrophic bacteria, “*Candidatus* Methylomirabilis,” oxidize CH_4_ in combination with nitrite reduction, while methanotrophic archaea, *Methanoperedens nitroreducens,* couple anaerobic CH_4_ oxidation with nitrate reduction (28, 29). Land use changes that affect soil hydrology, such as the drainage of waterlogged soil, can inhibit methanogenesis and complete denitrification that thrives in anaerobic and lower redox potential conditions of deep peat layers (30). Nitrification and incomplete denitrification can be enhanced in partially aerated soils, leading to higher N_2_O production (30). Furthermore, the close link between microbial carbon and nitrogen cycles and GHG fluxes emphasizes the importance of studying these processes together, particularly in relation to plant interactions (31).

Soil harbors a variety of microorganisms that are involved in the nitrogen cycle (23). Another potent GHG, N_2_O, is produced through nitrification, denitrification, and dissimilatory nitrate reduction of ammonia (DNRA), while N_2_O consumption is mediated by microorganisms that possess N_2_O reductase enzymes (23, 32). Anthropogenic activities, especially increased nitrogen inputs from fertilizer runoff, can exacerbate N_2_O emissions in plantations (33). In addition, peat characteristics and vegetation influence microorganisms and biogeochemical processes (34). There are some studies investigating microbial metabolic pathways in tropical peatland ecosystems in Southeast Asia (18, 35). These microbial communities were primarily analyzed by amplicon sequencing (36–39). Nonetheless, our understanding of the temporal changes in the microbiome and the associated genes encoding enzymes that regulate CH_4_ and N_2_O fluxes in tropical peatlands is limited. In particular, there are no studies on the temporal composition of the microbiome in tropical peatlands under different land uses in Sarawak, Borneo, the world’s third-largest island in Southeast Asia.

**Fig 1:**
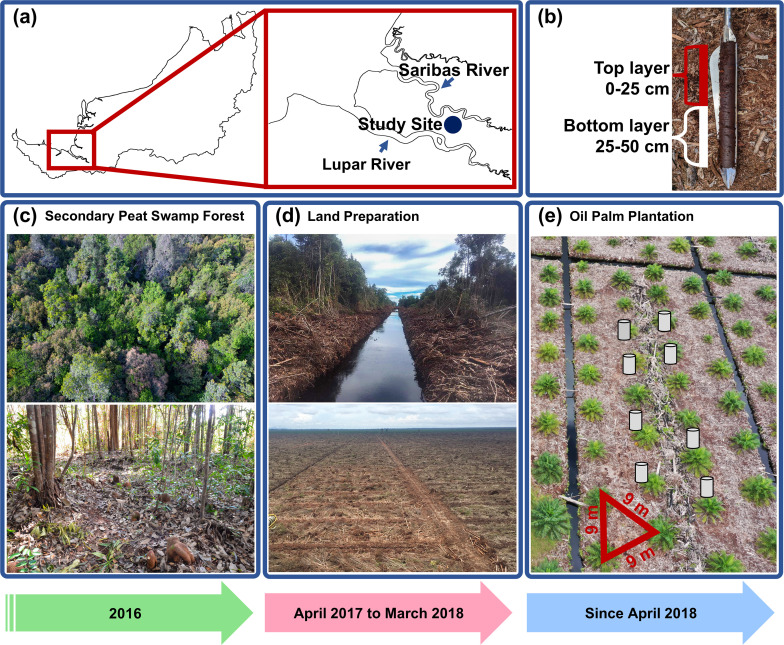
Study site location and land-use changes. Sampling at 1° 23’ 31.9” N 111° 23’ 49.1” E (a). Peat sampling depth (b). Logged-over secondary peat swamp forest aerial and ground-level view (c). The study site has undergone land preparation with canal construction for drainage (April 2017) and subsequent land clearing (d). Aerial view of the oil palm plantation in 2020; triangular crop spacing (red); a schematic representation of closed-chamber installation (gray cylinder) (e).

Our study presents data collected from 2016 to 2020, documenting temporal changes in land use at the same location in the tropics. We tracked the transition from secondary peat swamp forest through land preparation to the early stages of oil palm plantation (Fig. 1). This approach mitigates the typical spatial variability observed across sites in microbiome studies. The study site in Sri Aman, Sarawak (Fig. 1a) was originally a forest dominated by *Shorea albida* trees, which were selectively logged until commercial logging licenses were terminated in the 1980s. This cessation allowed the area to develop naturally into a secondary peat swamp forest characterized by *Litsea* spp. trees (Fig. 1c) (40). Land preparation began in April 2017 with the construction of drains to reduce the groundwater table and subsequent land clearing (Fig. 1d). By April 2018, the oil palm plantation was established with the planting of 1-year-old oil palm seedlings (Fig. 1e). Continuous sampling of the same site provided unique data sets on the soil microbiome and GHG measurements across different land use stages in tropical peatland.

The aims of this study are as follows: (i) to discover temporal changes in the microbial community during land use change, (ii) to uncover changes in microbial carbon and nitrogen cycles governing CH_4_ and N_2_O emissions due to land use change, and (iii) to reveal the greenhouse gas potential based on the microbiome and emissions during the transition of land use change in tropical peatland. We hypothesized that differences in GHG emissions are related to microbiome composition and functional gene abundances, and soil properties play a role in regulating genes associated with CH_4_ and N_2_O production and consumption.

## RESULTS

### Groundwater table, environmental variables, and peat chemical properties

**Fig 2:**
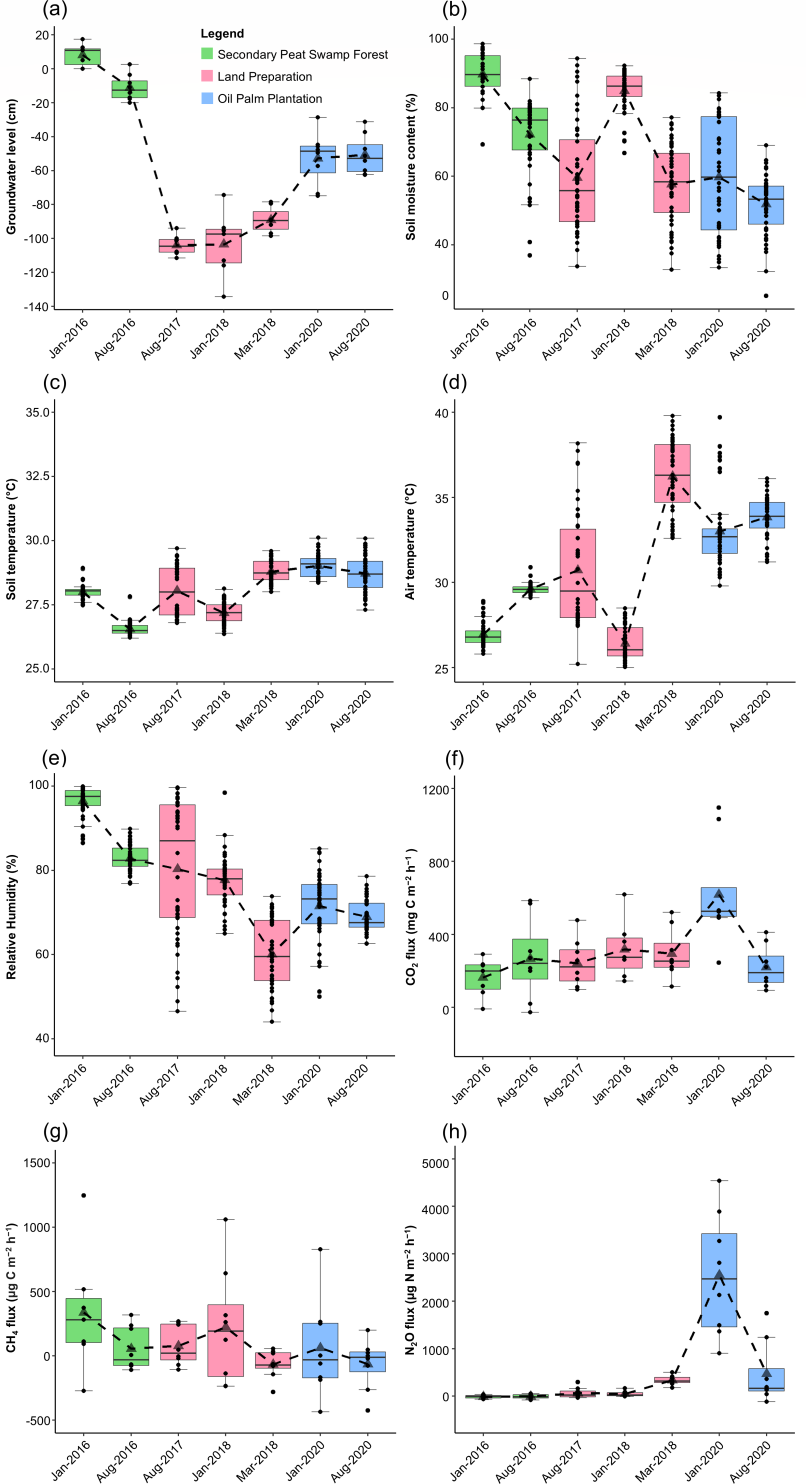
Variation in groundwater level (a), soil moisture content (b), soil temperature (c), air temperature (d), relative humidity (e), soil CO_2_ (f), CH_4_ (g), and N_2_O (h) fluxes. Dashed lines intercept the means (triangular point). Negative groundwater levels indicate water level below the surface. Negative emission values indicate a net uptake of respective GHG. The boxplots showed the interquartile range between the upper quartile (Q3) and the lower quartile (Q1) with the median (Q2, horizontal lines in the boxes) representing the data spread. The bottom whiskers represent the minimum [Q1–1.5(Q3–Q1)] and top whiskers represent the maximum [Q3 + 1.5(Q3–Q1)] limits. Outliers fall outside the limits of the whiskers.

Groundwater levels in the secondary peat swamp forest fluctuated between –11.1 and 8.5 cm in relation to the peat surface (Fig. 2a). The negative value signifies that the water levels were below the peat surface. After the construction of artificial canals and drainage systems in the land preparation, the groundwater level dropped to –104 cm (Fig. 2a). In the oil palm plantation, the water level was maintained within –50 cm (Fig. 2a). These changes in the groundwater table due to land use change and seasonal variations were significantly different (*P* < 0.05; Table S1).

Soil moisture content was the highest in the forest and averaged above 50% in the oil palm plantation (Fig. 2b; Table S1). Soil and air temperatures were lower in the forest and increased in the land preparation and oil palm plantation (Fig. 2c and d; Table S1). Relative humidity was above 80% in the forest and decreased as land use changed to an oil palm plantation (Fig. 2e; Table S1). These changes in environmental variables were significantly different due to land use change (*P* < 0.05; Table S1).

The conversion of forest to plantation influenced the humification level, total carbon, and concentrations of ammonium, nitrate, and phosphate, despite minimal change in pH values (Table 1; Table S2). The pyrophosphate solubility index (PSI), which measures the degree of humification of the peat, was lowest in the forest, followed by land preparation and oil palm plantation. A higher PSI value indicates a higher humification level (41). Total carbon was slightly higher in the oil palm plantation than in the forest. The inorganic nitrogen pool in the forest ecosystem was primarily composed of ammonium. The trend reversed, with nitrate levels peaking as the site transitioned to land preparation and then decreasing in the oil palm plantation. In addition, soil phosphate concentrations were lower in the oil palm plantation than in the land preparation and the forest.

**TABLE 1:** Peat chemical properties according to land use, seasonal variation, and sampling depth.^*a*^^,*b*^

Land use	Secondary peat swamp forest				Land preparation						Oil palm plantation			
Sample	Jan- 2016_T	Jan- 2016_B	Aug- 2016_T	Aug- 2016_B	Aug- 2017_T	Aug- 2017_B	Jan- 2018_T	Jan- 2018_B	Mar- 2018_T	Mar- 2018_B	Jan- 2020_T	Jan- 2020_B	Aug- 2020_T	Aug- 2020_B
Season	Wet	Wet	Dry	Dry	Dry	Dry	Wet	Wet	Wet	Wet	Wet	Wet	Dry	Dry
Depth (cm)	0­−25	25–50	0–25	25–50	0–25	25–50	0–25	25–50	0–25	25–50	0–25	25–50	0–25	25–50
pH	3.42 ± 0.1a	3.38 ± 0.1a	3.39 ± 0.0a	3.41 ± 0.0a	3.42 ± 0.0a	3.25 ± 0.1a	3.42 ± 0.0a	3.36 ± 0.2a	3.46 ± 0.0a	3.27 ± 0.2a	3.36 ± 0.0a	3.36 ± 0.0a	3.28 ± 0.0a	3.25 ± 0.1a
PSI	6.1 ± 0.8f	9.8 ± 0.3def	9.7 ± 0.8ef	16.3 ± 1.7bcde	10.1 ± 1.5def	15.1 ± 0.5cde	15.3 ± 1.2bcd	20.9 ± 0.1ab	19.1 ± 0.9bcd	25.2 ± 3.6ab	19.5 ± 0.1abc	22.4 ± 0.0abc	20.5 ± 1.4abc	25.4 ± 1.0a
Total C (%)	52.2 ± 0.7c	54.7 ± 0.8abc	53.1 ± 0.1bc	55.0 ± 0.3abc	52.5 ± 0.1c	54.8 ± 0.0abc	54.5 ± 0.6abc	56.1 ± 0.4ab	53.5 ± 0.2abc	54.5 ± 0.3ab	53.9 ± 0.1abc	54.5 ± 0.3a	54.6 ± 0.6abc	56.0 ± 0.0a
Total N (%)	1.9 ± 0.0a	1.7 ± 0.3a	1.9 ± 0.0a	1.7 ± 0.1a	1.9 ± 0.0a	1.8 ± 0.1a	2.0 ± 0.0a	1.8 ± 0.1a	1.7 ± 0.0a	1.6 ± 0.1a	1.8 ± 0.2a	1.7 ± 0.1a	1.8 ± 0.1a	1.7 ± 0.1a
C:N ratio	27.0 ± 0.0a	32.2 ± 5.3a	27.5 ± 0.1a	31.5 ± 1.8a	27.1 ± 0.1a	31.1 ± 1.3a	27.6 ± 0.7a	31.9 ± 1.3a	30.8 ± 0.5a	33.5 ± 1.9a	30.0 ± 2.8a	33.9 ± 2.8a	30.3 ± 2.0a	33.5 ± 1.6a
NO_3_^−^ (µg g^−1^ dry weight soil)	4.5 ± 0.0b	5.2 ± 0.5b	3.0 ± 0.7b	3.8 ± 0.2b	4.2 ± 0.0b	31.2 ± 0.0b	14.6 ± 13.2b	17.7 ± 3.4b	184.2 ± 43.6a	180.5 ± 17.1a	93.5 ± 34.8ab	48.9 ± 31.3b	89.0 ± 27.7ab	33.7 ± 1.0b
NH_4_^+^ (µg g^−1^ dry weight soil)	79.7 ± 10.8ab	40.6 ± 1.2abcd	78.8 ± 3.0abc	38.6 ± 1.2abcd	84.4 ± 6.3a	37.9 ± 18.4abcd	57.1 ± 17.7abcd	33.8 ± 12.9d	66.1 ± 11.5abcd	31.5 ± 3.3d	31.7 ± 7.2cd	31.9 ± 2.4cd	38.2 ± 6.5abcd	32.6 ± 2.0bcd
PO_4_^−3^ (µg g^−1^ dry weight soil)	441.0 ± 9.5ab	153.8 ± 0.1ab	410.9 ± 27.9ab	181.4 ± 23.4cd	466.6 ± 75.9a	200.8 ± 17.8cd	283.7 ± 13.3bc	169.7 ± 64.3cd	413.9 ± 10.9ab	194.9 ± 80.7cd	194.3 ± 42.6cd	112.9 ± 19.0d	153.3 ± 46.9cd	93.0 ± 18.4d

^*a*^ Values are mean ± SD (*n* = 2), followed by different lowercase letters to indicate significant differences at *P* < 0.05.

^*b*^ Top sample = T; bottom sample = B; total carbon = total C; total nitrogen = total N; nitrate = NO_3_^-^; ammonium = NH_4_^+^; phosphate = PO_4_^-3^.

No seasonal variation was found in the chemical properties based on the current samples (Table S2; *P* > 0.05). In addition, the peat chemical properties differ based on sampling depth (Table 1; Table S2). The PSI, total carbon, and C:N ratios were higher in the deeper peat layer (25–50 cm below the surface; Table 1). In contrast, total nitrogen and phosphate were higher in the top peat layer (0–25 cm below the surface; Table 1).

### Soil greenhouse gases

Soil CO_2_, CH_4_, and N_2_O fluxes varied across the different land uses (Fig. 2). The CO_2_ emissions increased significantly due to land use (*P* = 0.008) and seasonal variation (*P* = 0.020), with the highest value recorded during the wet season in the oil palm plantation (Fig. 2f; Table S1). Although not statistically significant, the highest CH_4_ emissions were observed in the wet season when the secondary peat swamp forest was waterlogged (Fig. 2g). The site remained a net CH_4_ source during the land preparation and the oil palm plantation phases.

The oil palm plantation N_2_O emissions were significantly higher than the secondary forest and land preparation (Fig. 2h; Table S1). The N_2_O emissions also showed seasonal variations, with the highest values occurring during the wet season in the oil palm plantation. Initially, the secondary peat swamp forest acted as a net N_2_O sink. However, when land clearing activities began, the site became a net N_2_O source, specifically as the site converted into an oil palm plantation.

### Prokaryotic communities structure and diversity in tropical peatland

The coverage and characteristics of the shotgun metagenomic sequencing data are detailed in Tables S3 to S5. The tropical peatland microbiota constituted mainly *Bacteria* (69%–79%), followed by *Eukaryota* (20%–29%), mainly *Arthropoda*, *Chordata*, and *Streptophyta*. Fungal DNA accounted for 8%–9% of the eukaryotic DNA, dominated by *Ascomycota* and *Basidiomycota*. *Archaea* constituted less than 3% of the total reads, while viruses, represented by *Uroviricota*, *Taleaviricota*, and *Artverviricota*, contributed 0.4%.

Coverage estimates and *Nonpareil* sequence diversity revealed that the diversity of the microbiome was lower in the oil palm plantation compared to the forest (Table S5; Fig. S2 and S3). However, the microbiota showed similarities in the forest, land preparation, and plantation, with *Proteobacteria*, *Actinobacteria*, and *Acidobacteria* dominating (Table S6; Fig. S4). Land use change significantly influenced the relative abundance of *Verrucomicrobia* (*P* = 0.012), while seasonal variation shaped *Planctomyces* populations (*P* = 0.014). In addition, peat depth played a role in the relative abundance of *Proteobacteria*, *Actinobacteria*, *Firmicutes*, *Bacteroidetes*, *Cyanobacteria*, and *Chloroflexi* (Table S6).

**Fig 3:**
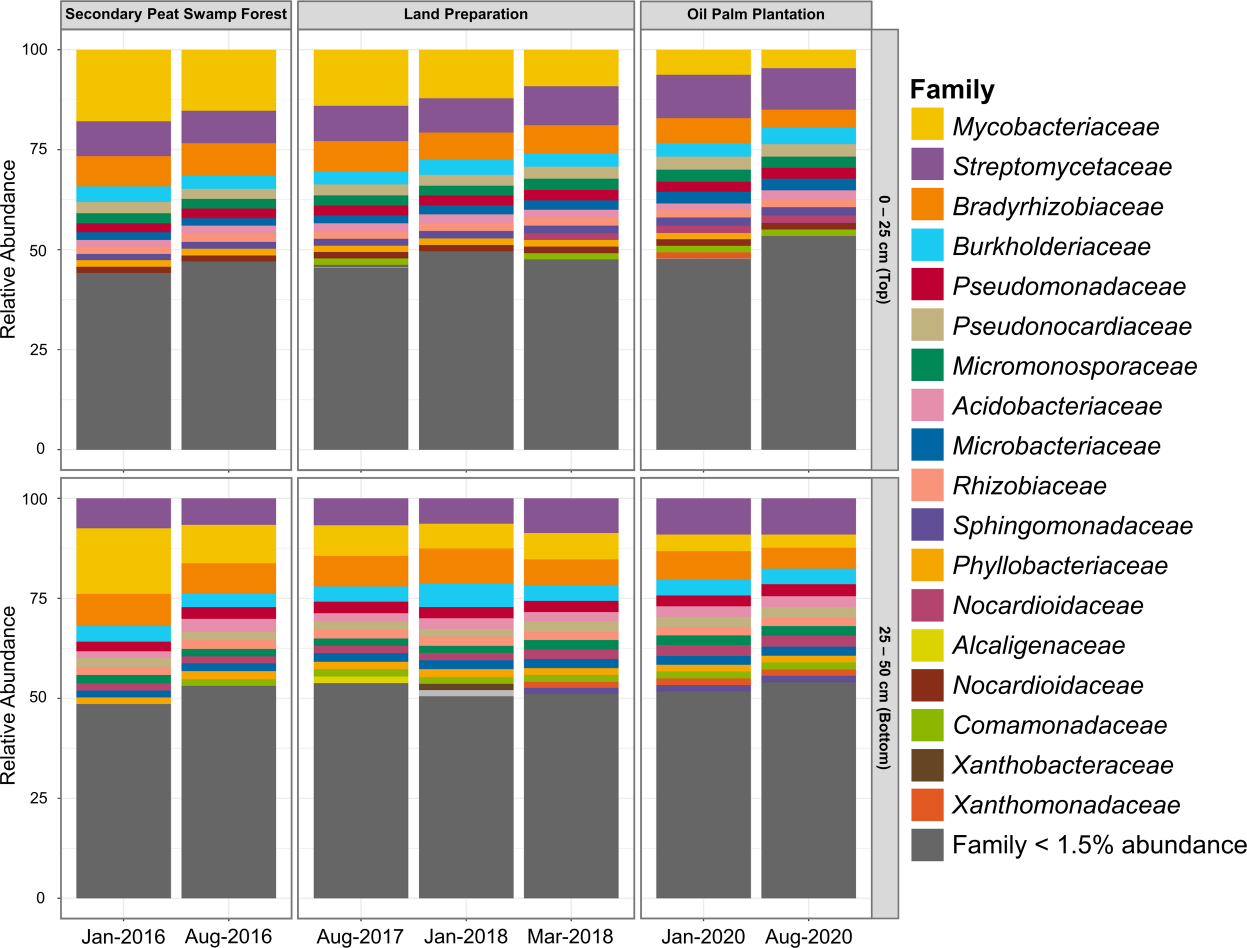
Relative abundance of tropical peatland prokaryotic families across temporal land use and sampling depth. Taxonomic families with less than 1.5% relative abundance were grouped.

The prokaryotic communities were dominated by the relatively abundant families from the phyla *Proteobacteria*, *Actinobacteria*, and *Acidobacteria* (Fig. 3). In particular, the transition from forest to plantation led to a decrease in the relative abundance of the families *Bradyrhizobiaceae*, *Mycobacteriaceae*, and *Streptomycetaceae*. The family *Acidobacteriaceae*, which dominates within *Acidobacteria*, remained predominant and was unaffected by peat depth.

The archaeal community was dominated by members of the phyla *Euryarchaeota*, “*Candidatus* Thermoplasmatota” and *Thaumarchaeota*, with significant differences in relative abundance due to land use change (Table S6). Several metanogenic taxa were identified, including *Methanobacteriales*, *Methanocellales*, *Methanococcales*, *Methanoliparales*, *Methanomassilicoccales*, *Methanomicrobiales*, *Methanonatronarchaeales*, and *Methanosarcinales*. Furthermore, the *Thaumarchaeota* also includes ammonia-oxidizing archaea (AOA) belonging to the class *Nitrososphaeria*.

### Prokaryotic community variation in response to abiotic factors

**Fig 4:**
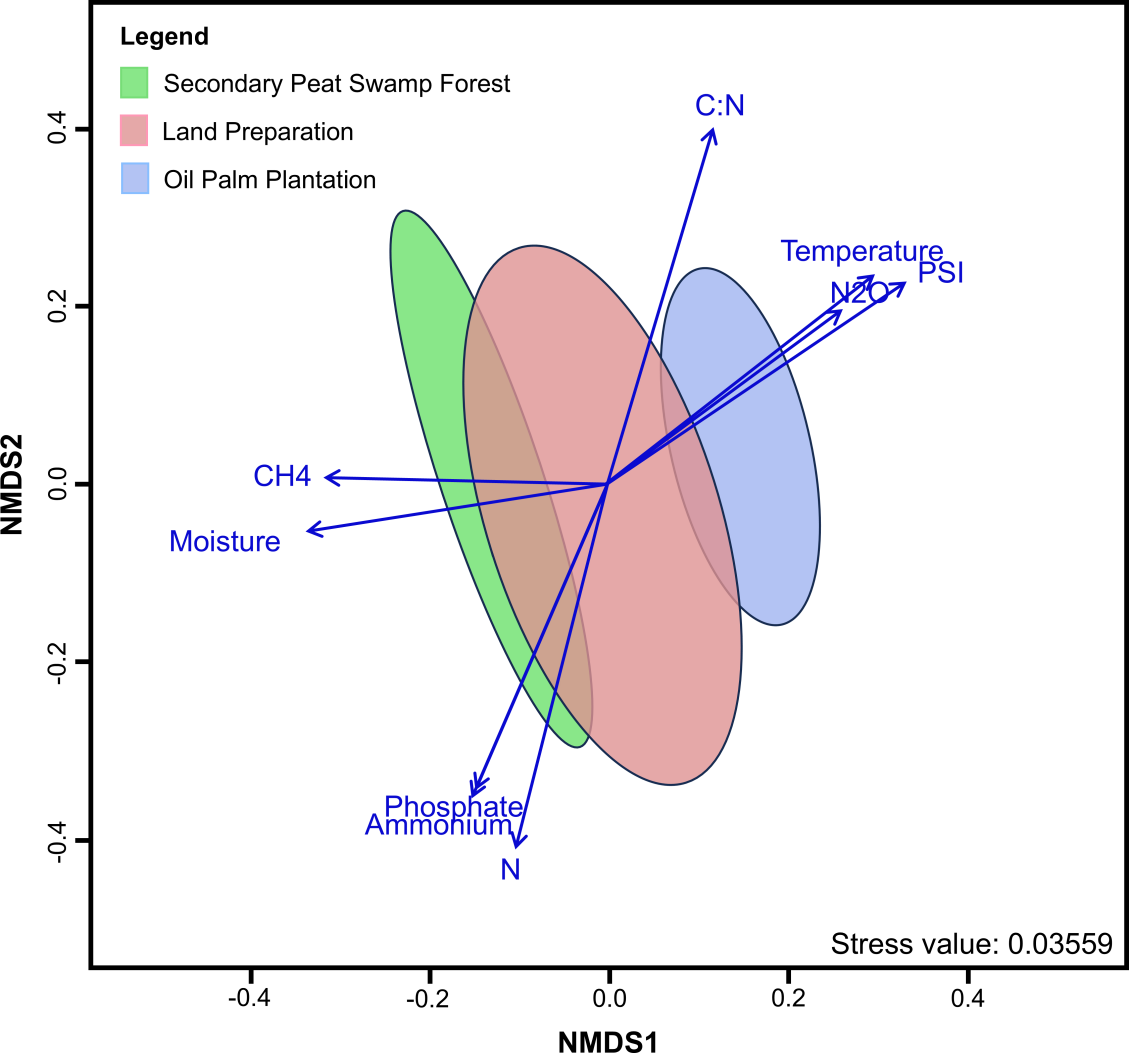
The non-metric multidimensional scaling (NMDS) plot of prokaryotic composition is based on the Bray-Curtis distance matrix. The peat samples’ chemical properties and GHG levels were fitted onto the NMDS ordination. Only significant factors that drive the prokaryotes are shown with *P < 0.05.* The arrow lengths are proportional to the strength of the correlation measured between peat chemical properties and each ordination axis. The ellipses represent the SD from the mean of the prokaryotic communities. The “green” ellipse represents the secondary peat swamp forest, with “red” and “blue” ellipses representing the land preparation stage and oil palm plantation, respectively. Stress value of 0.03559 indicated a good fit for the NMDS ordination summary of observed distances among prokaryotic communities.

The prokaryotic communities differed in response to land use change (Tables S7 and S8). Microbial community composition was more similar between forest and land preparation samples, with the greatest differences observed between the forest and plantation phases (Fig. 4). The ordination plot showed that prokaryotic communities transitioning more recently retained similarities to the initial land use. The low groundwater levels in the plantation potentially influenced humification levels, as shown by the higher PSI values. Changes in microbial composition were also associated with variations in the C:N ratio, total nitrogen, and N_2_O emissions. Through Mantel analyses, humification level showed the strongest correlation with prokaryotic diversity (Table S9). Other factors, including concentrations of ammonium and phosphate, groundwater level, pH, and C:N ratio, also showed a significant correlation with prokaryote diversity (Table S9).

### CAZymes analysis

**Fig 5:**
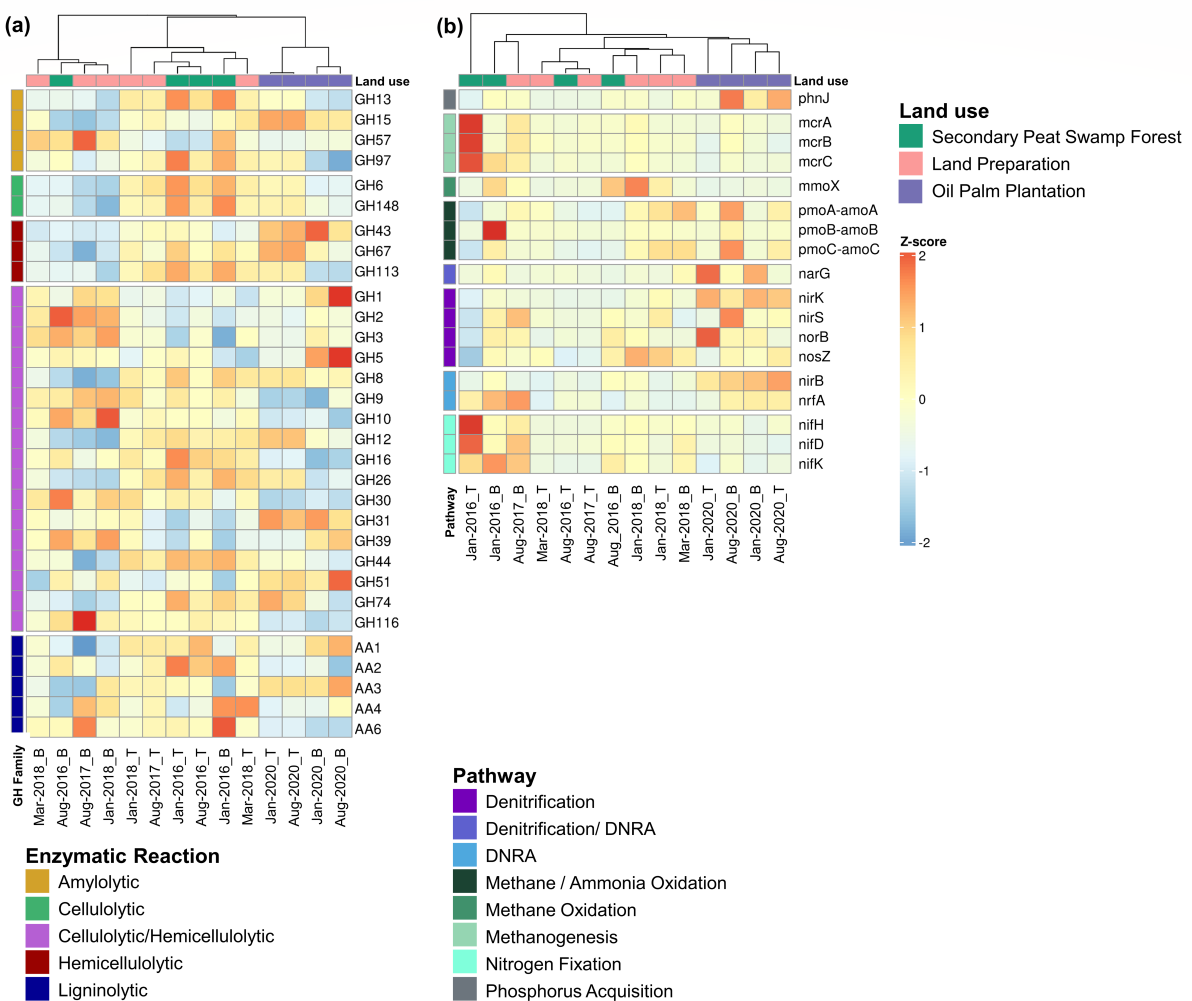
Heatmaps showing cluster analysis of tropical peatland functional potential. Distribution of selected CAZymes in the GH family and AA related to amylolytic, cellulolytic, hemicellulolytic, and ligninolytic enzymes (a). Soil respiration genes and pathways related to microbial production and consumption of CH_4_ and N_2_O (b). Heatmaps are row-scaled to *Z*-score ranging from two SDs below the mean value (−2, blue) to two SDs above the mean value (2, red).

The assembly statistics and the functional classification of the Cluster of Orthologous (COGs) are provided in Table S10; Fig. S5, respectively. The analysis of CAZymes highlighted the differences in carbohydrate processing, particularly in the decomposition of plant litter, which involves the breakdown of lignin, cellulose, and hemicellulose by different enzymes. In Fig. 5a, the CAZymes heatmap is categorized by selected glycoside hydrolase (GH) and auxiliary activity (AA) families. Amylolytic enzymes from the GH families 13, 15, 57, and 97, which act on glucosidic bonds in starch or short oligosaccharides, were identified.

Furthermore, cellulolytic enzymes from GH6 and GH148, including hemicellulosic enzymes from GH43, GH67, and GH113, were detected. These enzymes were present in the expected range in the upper layer of forest and land preparation peat samples; however, they were particularly scarce in the lower layer. Specific GH groups that include a combination of cellulolytic and hemicellulosic enzymes, such as GH1, GH2, GH3, GH5, and others, were distinguished on the heatmap (Fig. 5a).

The analysis also revealed the presence of AA families targeting lignin. Hemicellulolytic and ligninolytic enzymes acting on recalcitrant peat components were widespread in disturbed peat (land preparation and oil palm plantation). However, the plantation samples cluster separately from the forest and land preparation. Notably, sequences associated with GH1 and GH5 are also prevalent in the plantation samples.

### Microbial methane-cycling functional genes

**Fig 6:**
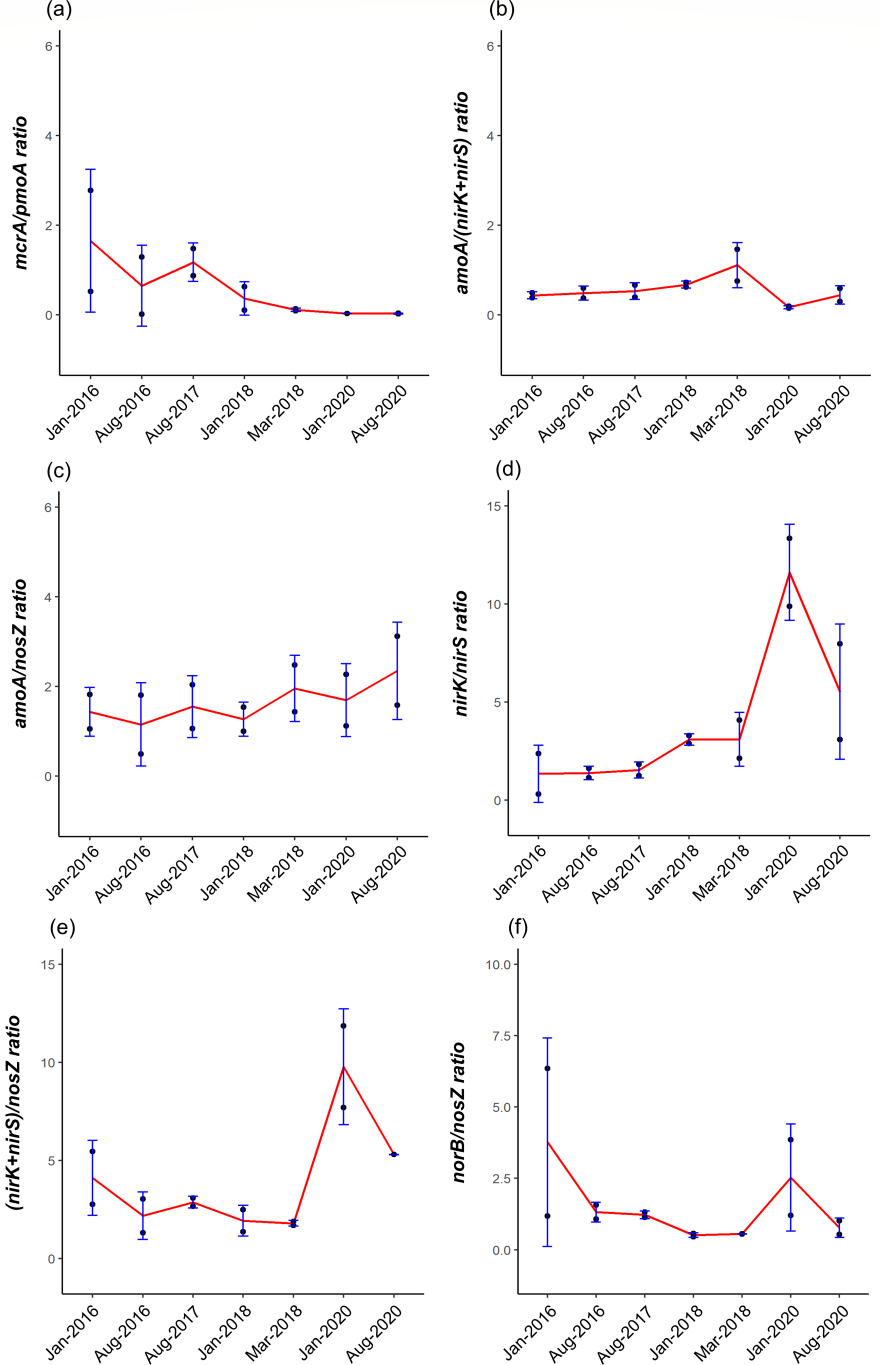
Ratio of methanogens (*mcrA*) to CH_4_ oxidizers (*pmoA*) (a), nitrifiers (*amoA*) to denitrifiers (*nirK* and *nirS*) (b), nitrifiers (*amoA*) to N_2_O reducer (*nosZ*) (c), nitrite reducers *nirK-*type to *nirS-*type (d), *nirK* and *nirS* to *nosZ* (e), and nitric oxide reducers (*norB*) to *nosZ* (f) in different land uses. The red lines intercept the mean of the respective gene ratio. The blue error bars represent the standard deviation.

Microbial methane-cycling functional genes were analyzed primarily through gene-based approaches, as detailed in the Materials and Methods, with metagenome-assembled genomes (MAGs) employed selectively to complement these analyses. The heatmap and cluster analysis of GHGs production and consumption revealed distinct patterns across land uses (Fig. 5b). Plantation samples clustered separately. In contrast, forest and land preparation samples exhibited comparable functional potential profiles. The *mcrA* gene abundance was higher in waterlogged forests (Fig. 6a), with major methanogens identified were *Methanocellales* and *Methanosarcinales* (Table S13). We also recovered MAGs encoding *mcrA*, assigned to the phylum *Halobacteriota* (GTDB classification), linked to *Methanocellales* and *Methanosarcinales*, thereby reinforcing and supporting the gene-centric analyses. In addition, genes related to CH_4_ production (*mcrABC*) showed significant correlations with PSI, C:N ratio, and CO_2_ fluxes (Table S11). Non-methanogenic CH_4_ production through phosphonate (*phnJ*) demethylation was also observed across land uses (Fig. 5b).

The α-subunit copper-containing membrane-bound particulate CH_4_ monooxygenase, encoded by the *pmoA* gene, was the predominant methanotrophic trait observed in our samples. Methanotrophic *Alphaproteobacteria*, particularly genera such as *Methylocystis*, *Methylosinus*, and *Bradyrhizobium*, were identified in all land uses and potentially CH_4_ regulators in tropical peatlands (Table S13). Importantly, the binning of *Methylocystis* MAGs provided further evidence supporting CH_4_ oxidation capabilities, underscoring the findings from our gene-centric analyses. In addition, *pmoA* genes were found in *Gammaproteobacteria* (mainly *Methylococcales*) and *Verrucomicrobia*. Genes involved in CH_4_ oxidation (*pmoABC*) were correlated with groundwater level, CH_4_ fluxes, total nitrogen, C:N ratio, ammonium, and phosphate levels (Table S11).

For the α-subunit iron-containing cytoplasmic soluble CH_4_ monooxygenase, encoded by the *mmoX* genes, was found in *Alphaproteobacteria*, *Gammaproteobacteria*, and *Actinobacteria*. The *mmoX* genes showed weak correlations with total nitrogen, C:N ratio, and concentration of ammonium and phosphate (Table S11). This study also highlighted the putative role of anaerobic oxidation of CH_4_ by archaea and bacteria, as these taxa were present in the 50 cm peat depth. Potential anaerobic CH_4_ oxidizers were the anaerobic methanotrophic archaea (ANME) members (“*Candidatus* Methanohalobium,” *Methanoperedenans*) and NC10 methanotrophic bacteria (“*Candidatus* Methylomirabilis”). Only *Methylomirabilota* MAGs were recovered for the data sets, while MAGs for ANME members, such as “*Candidatus* Methanohalobium” and *Methanoperedenans*, were not detected.

### Microbial nitrogen-cycling functional genes

The gene-centric analyses identified sequences that may play key roles in nitrification, primarily driven by AOA and ammonia-oxidizing bacteria (AOB), integral to the nitrogen cycle. The dominant AOA was *Thaumarchaeota* (class *Nitrosophaeria*), detected and found across all land uses, while most AOB were *Proteobacteria*. The *amoA* genes exceeded the nitrite reductase (*nir*) genes abundance in the later stage of the land preparation, suggesting conditions that favor ammonium-oxidizing microorganisms (Fig. 6b). Ammonium oxidation genes were correlated to groundwater levels and CH_4_ fluxes (Table S12). Nitrification also likely contributes to N_2_O production in the oil palm plantation, as evidenced by a threefold increase in the *amoA* to *nosZ* ratio (Fig. 6c). Furthermore, *Nitrosotalea* MAGs encoding the *amoABC* were recovered, suggesting their possible role in nitrification and their significance in the nitrogen cycle.

In the oil palm plantation, the *nirK* genes exceeded *nirS* count by 3–13-fold (Fig. 6d). Across different land uses, the relative abundance of *nirK* genes was higher than *nirS*, with *nirS* exceeding *nirK* gene abundance only in the top layer January 2016 forest sample, indicating dominant *nirK*-type denitrification in tropical peatlands (Fig. 6d). The major genera of *nirK*-type denitrifiers were *Bradyrhizobium*, *Pseudomonas*, *Mesorhizobium*, *Rhizobium*, *Rhodopseudomonas*, *Bosea*, *Paracoccus*, *Achromobacter*, *Sinorhizobium*, and *Ensifer* (Fig. S6a). Mantel analyses of *nir* genes showed correlations with groundwater levels, CO_2_, and N_2_O fluxes (Table S12).

The direct source of N_2_O, facilitated by *norB*, showed a higher gene abundance in deeper peat layers. The *norB-*encoding prokaryotes belong to members of *Proteobacteria* (*Burkholderia* and *Ralstonia*), *Acidobacteria* (*Terriglobus* and *Paludibaculum*), *Actinobacteria* (*Nonomuraea*), and *Planctomycetes* (*Gemmataceae*; Fig. S6b). In addition, *Bacteroidetes*, *Chlamydiae*, *Chloroflexi*, *Cyanobacteria*, *Gemmatimonadetes*, *Nitrospirae*, *Spirochaetes*, and *Verrucomicrobia* also encoded *norB*, indicating broad microbial contribution to N_2_O production across land uses. Most MAGs recovered encoding *norB* genes were affiliated with *Pseudomonadota* and *Acidobacteriota*. Correlation analyses of *norB* genes indicated a weak correlation with total nitrogen and the C:N ratio (Table S12).

The *nosZ* gene, which mediates the final step of denitrification, is the only known biological process that converts N_2_O to N_2_. Taxa encoding complete denitrification genes, such as *Magnetospirilium*, *Ralstonia*, *Burkholderia*, *Paraburkholderia*, *Dyella*, and *Terriglobia* were more abundance during the land preparation and in the oil palm plantation (Table S13). Predominant taxa encoding the *nosZ* gene include members of the phyla *Proteobacteria* and *Acidobacteria*. In addition, the relative abundance of *Bradyrhizobium* and *Methylocystis* decreased as the forest transitioned to oil palm plantation (Fig. S7a). The ratio of nitrite reducers (sum of *nirK* and *nirS*) to N_2_O reducers (*nosZ*) was the highest in January 2020 of the oil palm plantation sample, increasing from 7- to 11-fold (Fig. 5e). The abundance of *norB* genes was three times higher than that of *nosZ* (Fig. 5f). The main N_2_O producers are possibly the *nirK*-type denitrifiers with gene abundance exceeding *nirS*-type denitrifiers. This coincides with the increase in N_2_O fluxes observed in January 2020 (Fig. 2h).

The fermentative DNRA (*nirB*) genes were more relatively abundant than the respiratory DNRA (*nrfA*). Members of *Proteobacteria* (order *Burkholderiales*, *Caulobacterales*, *Hyphomicrobiales*, *Lactobacillales*, *Methylococcales*, *Nevkiales*, *Pseudomonadales*, *Rhodospirilales*, and *Xanthomonadales*) encoded *nirB*-mediated DNRA. The *nrfA*-mediated DNRA was dominated by the class *Terriglobia* (*Acidobacteria*), with lower occurrence in the plantation samples. The DNRA pathway produced N_2_O as a by-product, and the lower abundance of DNRA genes in the oil palm plantation suggests a minor contribution to N_2_O emissions.

Metagenomic analysis also revealed a widespread presence of nitrogen fixation (*nif*) genes in archaea and bacteria. *Alphaproteobacteria* was the dominant nitrogen fixers in tropical peatlands, with minor contributions by *Beta-*, *Delta-*, and *Gammaproteobacteria*. The *nif* genes were also detected in *Acidobacteria*, *Actinobacteria*, *Bacteroidetes*, *Chlorobi*, *Chloroflexi*, *Nitrospirae*, *Planctomycetes*, and *Verrucomicrobia*, indicating broad community participation in nitrogen-fixing. The major taxonomic families of diazotrophs are shown in Fig. S7b, with *Bradyrhizobiaceae* decreasing when the site transitioned to an oil palm plantation. Overall, the forest samples from January and August 2016 had a higher abundance of *nifH* genes than the plantation (January and August 2020; Fig. S7b). In addition, *nifH* genes were detected in methanogens (i.e., *Methanomicrobium*, *Methanothrix*, *Methanocella*, *Methanoregula*, *Methanosarcina*, *Methanolinea*, and *Methanolobus*) and ANME members (“*Candidatus* Methanoperedens”), suggesting a possible coupling of nitrogen fixation to CH_4_ metabolism.

## DISCUSSION

Land development in tropical peatlands alters carbon and nitrogen cycles due to changes in vegetation, litter accumulation, and decomposition rate (42–44). In oil palm plantations, groundwater levels are deliberately lowered to approximately 50 cm, as oil palm feeder roots are most active at this depth for optimal crop growth and root development (45). Groundwater levels in oil palm plantations on tropical peatlands are managed through drains, canals, and water-blocking structures (weirs) for water retention and drainage (46). Adjustments to groundwater levels are made depending on the stage of oil palm development.

**Fig 7:**
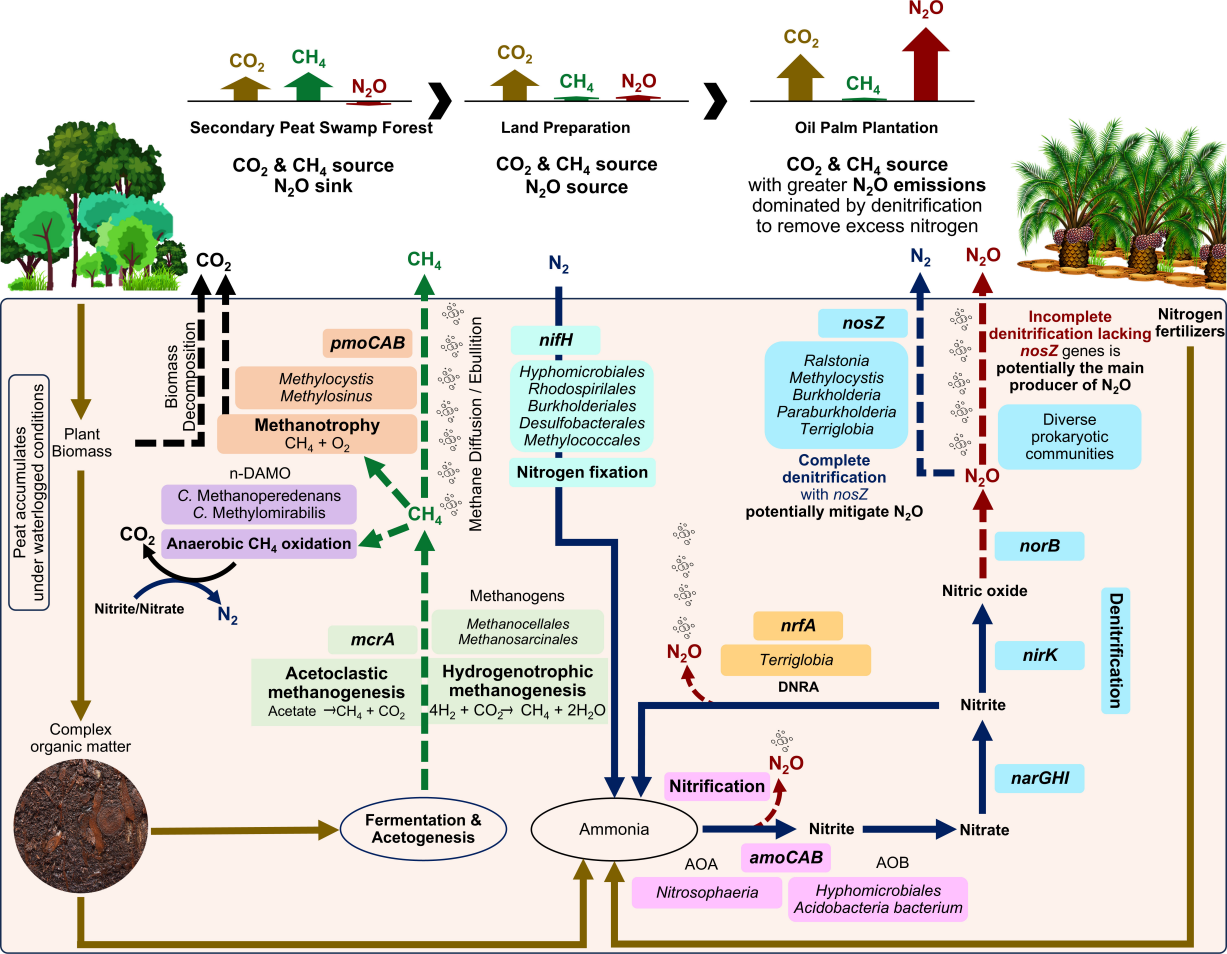
Major taxa, genes, and processes related to CH_4_ and N_2_O production and consumption, including nitrogen fixing in tropical peatland. Dashed arrows represent putative atmospheric GHGs emissions from peat. Important GHGs are CO_2_, CH_4_, and N_2_O. The GHGs fluxes between land uses were summarized in the schematic diagram. Naturally, CO_2_ is released through plant biomass decomposition in the secondary forest, with higher CO_2_ levels in the oil palm plantation. Forest samples have higher CH_4_ emissions produced by methanogens in waterlogged conditions. The plantation is characterized by higher N_2_O emissions produced directly or indirectly mainly through denitrification with diverse prokaryotic communities. Gene abundance suggests a minor contribution by nitrification and DNRA pathways.

In this study, the secondary peat swamp forest transitioned from being a net N_2_O sink and a source of CO_2_ and CH_4_ to a net GHG source with increased CO_2_ and N_2_O emissions during land preparation and in the oil palm plantation (Fig. 2 and 7). Higher soil temperatures likely stimulate microbial activities that increase GHG emissions (47). As land use changes, the removal and alteration of aboveground vegetation affect humidity and temperature, while lowering the groundwater table increases the oxic layer, affecting peat decomposition and GHG fluxes (Table S1). Increased CO_2_ emissions have been attributed to heightened oxidative peat decomposition (48). High groundwater levels and moisture content could saturate peat layers and restrict aeration, increasing CH_4_ emissions (49). Nitrate levels possibly increased as a result of the mineralization of dead plant material after land clearing (Table 1). In addition, surplus nitrogen, primarily from nitrogen-based fertilizers, exceeds plant requirements and is transformed in the soil, increasing N_2_O fluxes in the oil palm plantation.

Long-term over-fertilization in the plantations can lead to soil acidification, reduce microbial diversity, and increase GHG emissions (33, 50, 51). Building on these known effects, our study investigates how land use changes affect prokaryotic communities and the functional potential of specific microbial groups. Additionally, this report addresses the underrepresentation of tropical peatland metagenomic studies compared to other climate zones.

### Land use change influences prokaryote composition

Microbial communities in recently transitioned soils often retain taxa similarities to those of their previous land uses (52). Over time, these communities gradually develop unique traits specific to the new land use. Our results showed that the microbial composition in the secondary peat swamp forest was more similar to the land preparation phase than in the oil palm plantation. This suggests that while microbial community changes begin during the land preparation phase, more pronounced shifts occur with the introduction of oil palm seedlings and fertilization. The rhizosphere of young oil palms and the influx of nutrients from fertilization probably lead to significant shifts in microbial population structure and functions (17, 53).

The predominant bacterial phyla in all three land use types*—Proteobacteria*, *Actinobacteria*, and *Acidobacteria*—as well as the archaeal phyla *Euryarchaeota* and “*Candidatus* Thermoplasmatota” (Fig. S4), are consistent with reports from other studies on tropical peatland microbiomes (23, 36, 39). *Proteobacteria*, the most abundant taxa, play a key role in carbon and nitrogen cycling, while *Actinobacteria* is essential plant decomposers (36, 54). *Acidobacteria* can survive in various oxygen gradients, utilize different carbohydrates and nitrogen sources, and are well suited for nutrient-limited peatlands (55). In boreal and temperate peatlands, *Acidobacteria* are more dominant than *Proteobacteria* (56). However, *Acidobacteria* ecological traits remained largely undescribed and individual clades can adapt to different habitats (57).

### Microbial CH_4_ production and oxidation in tropical peatland

This study identified core taxa and the putative primary biogeochemical processes governing CH_4_ and N_2_O in tropical peatlands (Fig. 7), which include *Proteobacteria*, *Acidobacteria*, *Euryarchaeota*, and *Thaumarchaeota*. Waterlogged and anaerobic conditions in the secondary peat swamp forest likely contributed to higher CH_4_ emissions (Fig. 2). Methanogenesis involves diverse microbial groups with substrates supplied through fermentation and acetogenesis. *Methanomicrobia*, specifically the orders *Methanocellales* and *Methanosarcinales*, were the main methanogens (Fig. 7). However, CH_4_ fluxes in tropical peatland can vary throughout the year and are influenced by vegetation, groundwater levels, and nutrient levels (13).

Although CH_4_ and N_2_O concentrations are lower than CO_2_, they are more potent GHGs per molecule, which is a concern in disturbed soils (26, 32). Most CH_4_ is produced through two main pathways, acetoclastic and hydrogenotrophic methanogenesis, depending on substrate availability (58). Dominant *Methanocellales* in the secondary peat swamp forest ecosystem suggests that hydrogenotrophic methanogenesis is the primary source of CH_4_ in this environment. The hydrogenotrophic methanogenesis is favored as it produces more energy than acetoclastic methanogenesis in nutrient-limited conditions (59, 60). Moreover, methanogens can couple this process with nitrogen fixation, providing an alternative source for nitrogen in anoxic conditions (27). However, methanogenesis can also be outcompeted for substrates by sulfur-reducing bacteria (i.e., acetate, hydrogen, and CO_2_), which could suppress CH_4_ emissions (61). Although methanogens dominate CH_4_ production, facultative anaerobic wood-rot fungi have also been reported to emit CH_4_ through the halomethane-dependent pathway (62).

In our study, *Proteobacteria* and *Verrucomicrobia* dominated aerobic CH_4_ oxidation. Methanotrophic *Alphaproteobacteria*, especially the family *Methylocystaceae*, can be found across peatlands in the tropics (South America and Southeast Asia) and boreal regions (North America) (36, 63, 64). These ubiquitous *Methylocystaceae* are resilient to changes in the aboveground vegetation (65). As for anaerobic CH_4_ oxidation, *Methanoperedens nitroreducens* can convert CH_4_ to CO_2_ through reverse methanogenesis (66). This archaea then supplies nitrite to methanotrophic bacteria such as “*Candidatus* Methylomirabilis” to facilitate anaerobic CH_4_ oxidation (28, 67).

### Denitrification as primary N_2_O source in disturbed tropical peatland

A 2-year-old oil palm plantation in our study had higher CO_2_ and N_2_O emissions compared to previous land uses. The GHG fluxes aligned with plantation practices that lower groundwater levels and apply nitrogen-based fertilizers (e.g., NPK [nitrogen, phosphorus, and potassium] compound fertilizer, urea, ammonium chloride, ammonium nitrate, and ammonium sulfate) to promote root development, growth, and yield potential of young oil palms (33, 68). Higher nitrogen-based fertilization in mature oil palm plantations could lead to higher N_2_O emissions and peat decomposition (53). However, overall CO_2_ and N_2_O emissions from well-managed oil palm plantations can decrease over time through appropriate nutrient and water management strategies as the oil palms mature (53).

Nitrification initiated by AOA and AOB indirectly produces N_2_O (Fig. 7). Similar to our results, *Nitrososphaera*, which encodes the *amoA* genes, is dominant AOA in the low-nutrient acidic tropical peatlands (69). The AOA has a higher substrate affinity, which gives them a competitive advantage in environments with low ammonia concentrations (69). However, under conditions of increased nitrogen fertilization and liming, N_2_O emissions are likely by-product of nitrification by AOB nitrification as AOB emit higher levels of N_2_O than AOA (70, 71). Therefore, managed peatlands that utilized slow-released ammonia fertilizers may allow AOA to dominate nitrification, potentially resulting in lower N_2_O emissions.

Similar to nitrification, DNRA or nitrate ammonification is a minor contributor to N_2_O production based on gene relative abundance (Fig. 7). Lower DNRA gene abundance indicates that the ecosystem does not retain nitrogen, instead favors removing excess nitrogen through denitrification (35). In our study, denitrification appears as the primary source of N_2_O, evidenced by gene ratios and diverse *nirK-*type denitrifiers community identified. The *nirK* gene encodes for copper-containing nitrite reductase protein and elevated copper levels induced by copper fertilizer might favor *nirK*-type denitrifiers (72, 73). Higher *nirK*-type denitrifiers abundance and diversity than *nirS*-type have been observed in other natural and drained peatlands (23). Nitric oxide reductases (*norB*), part of the denitrification pathway, are the direct source of N_2_O and can also be found encoded by diverse bacterial communities (74). Although we could not detect the *norB* gene in archaea, complete denitrification pathways have been reported for *Haloarculaceae* and *Haloferacaceae* (75). The co-occurrence of denitrification genes in different prokaryotes emphasized the modularity of this pathway, in which intermediate molecules interact with other nitrogen metabolic pathways (76).

Incomplete denitrification without the *nosZ* gene was the major source of N_2_O in this study (Fig. 7). Increased N_2_O emissions were attributed to N_2_O production exceeding *nosZ* activities (Fig. 2 and 6). In nitrogen-rich environments, *Proteobacteria* and *Acidobacteria* are the predominant *nosZ*-encoding taxa (77). *Bradyrhizobium*, *Methylocystis*, *Ralstonia*, *Burkholderia*, *Paraburkholderia*, and *Terriglobus*, which encode *nosZ*, could reduce N_2_O emissions in tropical peatlands (Fig. S7a). Some non-denitrifiers also contribute to N_2_O reduction and encode only the *nosZ* genes without the *nir* or *norB* genes for energy conservation (76, 78).

The lower gene abundance of *nifH* in the oil palm plantation suggested potential suppression of diazotrophs (Fig. 5b). Diazotrophic bacteria such as *Bradyrhizobium* that encode *nosZ* have the potential to regulate soil N_2_O emissions (79). Therefore, suppression of *Bradyrhizobium* in the oil palm plantation can potentially affect N_2_O reduction to N_2_. Other diazotrophs, such as the oligotrophic *Geobacter* and *Anaeromyxobacter*, may also be suppressed in nutrient-rich environments (80). These oligotrophic taxa can be outcompeted by fast-growing copiotrophic taxa in long-term fertilization.

The forest site in this study, initially a net N_2_O sink, transitioned to a net N_2_O source as nitrate and humification levels increased in response to land clearing and conversion to an oil palm plantation. The *nir* genes positively correlated with N_2_O fluxes (Table S11). However, N_2_O emissions can vary across natural and managed ecosystems depending on factors such as groundwater table, soil carbon, and nitrogen availability (10, 81–83). Aerobic and anaerobic microsites near the soil surface in managed land with high carbon and nitrogen availability may promote coupled nitrification-denitrification reactions (82). Denitrification by *norB* occurs within these microsites with limited or no oxygen, influenced by rainfall and groundwater table fluctuation (84). Dominant *nirK*-type denitrifiers are more likely to perform incomplete denitrification, and consequently, the presence of higher *nir* to *nosZ* genes leads to higher N_2_O emissions (78). Nitrous oxide emissions also correlate positively with nitrogen fertilization rates (53, 85). Therefore, the oil palm industry can potentially mitigate N_2_O emissions by balancing nitrogen fertilization practices to reduce over-fertilization while still meeting plant nutrient requirements and by implementing strategies to minimize nitrogen losses through microbial denitrification.

The findings in this study highlight that disturbed tropical peatland ecosystems emit substantial GHGs driven by specific microbial groups and conditions. While these observations may be site specific, the study provides valuable insights into the dynamics of microbial composition and GHG emissions over the monitoring period from 2016 to 2020, considering specific ecosystem functions, environmental parameters, and peat chemical properties. The relative abundance of microbial composition may not fully capture changes in absolute abundance, and the predicted functions may not always align with active functions. Nonetheless, our approach offers a comprehensive assessment of tropical peatland microbial communities, covering a wide range of taxa and functional potential through reads and gene-based analyses. Future studies could benefit from incorporating multi-omics data to elucidate active biogeochemical processes and track changes in microbiome composition and functions in response to land use changes (52).

### Conclusion

This study investigated the impact of land use change on prokaryotic communities, peat chemistry, plant litter decomposition, GHG emissions, and ecosystem functioning. Prokaryotic communities correlated with humification levels, groundwater table, pH, C:N ratio, and concentration of ammonium and phosphate. Although CH_4_ fluxes from soil were negligible, *mcrA* genes associated with *Methanocellales* and *Methanosarcinales* were present across different land uses. The CH_4_ fluxes correlated with the groundwater table, humification levels, and the C:N ratio. Major CH_4_ oxidizers, particularly the *Methylocystis* group, which also encodes for *nosZ* genes, were negatively affected by land use changes, potentially influencing N_2_O regulation potential. Microbial community functional potential through gene abundance and the ratio of *nir* to *amoA* and *nosZ* suggests that N_2_O production was primarily driven by denitrification with minor contributions from nitrification. The N_2_O fluxes were correlated with the groundwater table, total nitrogen, and C:N ratio. Agricultural practices, such as lowering groundwater levels and fertilization, could stimulate denitrifying microbial communities. Land use changes have transformed the forest site from a CO_2_ and CH_4_ source and N_2_O sink to a source of CO_2_, CH_4_ and N_2_O following land preparation and oil palm cultivation. This study suggests that limiting soil carbon and nitrogen availability may be crucial for regulating microbial-mediated GHGs. While these findings shed light on the genetic potential of tropical peatland microbiome, further research is needed to validate the ecological contributions of less-common taxa and whether the inferred CH_4_ and N_2_O metabolic pathways are active. Ideally, long-term monitoring of tropical peatlands is essential to assess ecosystem resilience and inform sustainable management practices.

## MATERIALS AND METHODS

### Study site description

Fieldwork for this study was conducted from 2016 to 2020, covering the transition of land from a secondary peat swamp forest (Fig. 1c) to a cleared land prepared (Fig. 1d) for oil palm planting in a 9 × 9 m triangular pattern (Fig. 1e). Field sampling trips were conducted in months indicated in Table 1 to measure GHG emissions, associated peat chemical properties, environmental variables, microbial communities, and genetic potential affected by land use change.

The mean annual temperature is about 27°C, with the annual precipitation ranging from 2,734 to 3,312 mm (Fig. S1) during this period. Temperature and rainfall data were retrieved from the Department of Irrigation and Drainage of Malaysia in Sri Aman, Sarawak. Historically, the tropical peat swamp forest remained waterlogged throughout the years except during the dry season (May to September) when groundwater levels temporarily receded below the peat surface. The wet season, marked by heavy rainfall, peaks in January and spans from November to March (86).

Following land clearance, groundwater levels were artificially managed (lowered or raised) to support the establishment of oil palm plantations on tropical peatlands, using drains, canals, and water-blocking structures (weirs) (46). Chemical fertilizers for young oil palm trees were applied by the plantation management in two to three rounds a year, beginning in June 2018 and subsequent in April through May and September through October, avoiding months with high rainfall intensity or dry periods, with the amount adjusted based on palm age and crop requirement. Young palms received the following annual amount: nitrogen in the form of 1.0–2.0 kg of ammonium sulfate and 0.5–1.5 kg urea; phosphorus in 2.0–3.0 kg of rock phosphate; potassium in 1.5–2.5 kg muriate of potash; and micronutrients in 0.1–0.2 of copper, zinc, and borate.

### Soil respiration sampling

We sampled soil respiration (CO_2_, CH_4_, and N_2_O gases) using the closed-chamber method (13, 21, 33). At each sampling time, eight open-ended stainless-steel cylinders (25 cm height; 10 cm radius, *n* = 8) were randomly installed *in situ* during the different years as the land transitioned from a secondary peat swamp forest to the land preparation phase and then to an oil palm plantation (Fig. 1). In the secondary peat swamp forest and land preparation, the chambers were placed randomly within a 50 m radius. In the oil palm plantation, the chambers were installed 1.5 m from the base of the oil palm trunks (Fig. 1e).

For estimating CO_2_ soil respiration, 250 cm^3^ surface air was collected at zero minute, followed by another 250 cm^3^ air sampling from each closed chamber at 4-minute intervals. These samples were then transferred into Tedlar gas sampling bags using a 25 mL syringe connected to the lid of the closed chamber through silicone tubes. The linear relationship between CO_2_ efflux and time using the closed-chamber method has been independently validated in other tropical peatland studies, with sampling intervals of 4, 10, and 40 minutes demonstrating consistent proportional relationships (11, 13, 21, 33, 87). For CH_4_ and N_2_O gas, 20 cm^3^ of air samples was collected from each chamber at zero minute and subsequently after chamber closure at 10-, 20-, and 40-minute intervals (13, 33). The 20 cm^3^ air samples were transferred into pre-vacuumed gas chromatography (GC) vials and transported to the laboratory.

### Environmental variables, peat sampling, and groundwater measurement

Relative humidity and air temperature were measured using TESTO 625 (Testo SE & Co. KGaA, Germany). Soil temperature (10 cm depth) was measured using Checktemp 1 HI-98509 digital thermometer (Hanna Instruments, USA). Soil moisture level was measured using the Soil Moisture Meter DIK-311F (Daiki, Japan). These parameters were measured (in six replicates) in the vicinity of each chamber during soil respiration sampling. Perforated polyvinyl chloride (PVC) pipes were installed in auger holes, and groundwater levels were determined by subtracting the height of the pipes from measurements taken from the water surface to the top of the pipes using a measuring tape (*n* = 8 per sampling time).

The following procedure was used to collect composite peat samples from eight sampling points for chemical analyses and metagenome extraction during each sampling time. A peat auger (Eijkelkamp, The Netherlands) was used to collect peat samples (Fig. 1b), from which composite peat was extracted. Large woody materials were removed, and samples were divided based on depths: 0–25 cm (top-layer, represented by “T” in sample ID) and 25–50 cm (bottom-layer, represented by “B” in sample ID; Fig. 1b; Table 1). Each sampling point was augered in triplicates, divided, and then homogenized (*in situ*) to create composite samples of top and bottom layers. In each sampling time, two composite samples per depth were collected for chemical analysis. Similarly, for metagenome extraction, peat samples from all eight sampling points (multiple augering) were homogenized to form one large composite sample per depth. All pooled top- and bottom-layer samples were sealed in zip-locked bags and transported on ice. Upon arrival, samples for microbiome analysis were stored at −80°C until total DNA extraction. Peat samples for chemical analysis were air dried, sieved (2 mm), and stored at 4°C before analysis.

### Chemical analyses and greenhouse gas measurements

The pH, PSI, total C (%), total N (%), C:N ratio, nitrate (ppm), ammonium (ppm), and phosphate contents were determined in the soil samples using standard procedures (13). The CO_2_ gas concentration in the 250 cm^3^ Tedlar bags was measured within 6 hours after collection using an Infrared CO_2_ gas analyzer (Fuji Electric ZFPGC11, Japan) set up in the field. The gas vials with 20 cm^3^ air samples were transported back to the laboratory, and the CH_4_ gas concentration was measured using a gas chromatography system with a flame ionization detector (Agilent 7890A, USA). The N_2_O concentration was measured using a gas chromatography system with an electron capture detector (Agilent 7890A, USA). The CO_2_, CH_4_, and N_2_O gas fluxes were calculated based on the linear accumulation of gases with time in the closed chambers (13, 33). Additional information on peat chemical analyses and soil respiration measurements was described in Supplementary Information S1 and S2.

### DNA extraction and purification

Environmental DNA was extracted from 1.5 g of peat using FastDNA Spin Kit for Soil (MP Biomedical, USA) using three manufacturer’s microcentrifuge tubes, with each tube containing 0.5 g of peat. The samples were lysed using TissueLyser II (Qiagen, Germany) with bead beating at 30 Hz for 3 minutes, repeated five times with a minute on ice at intervals. Then, humic substances were removed using 500 µL of 5.5 M guanidine thiocyanate, and the DNA pellets were washed at least three times until the Binding Matrix beads returned to their original color. Further purification was done using Agencourt AMPure XP beads (Beckman Coulter Life Sciences, USA). Purified DNA was eluted with nuclease-free water. For each composite sample, high-quality purified DNA from the three extraction replicates was pooled to represent the samples accordingly. In total, the following numbers of samples were prepared for metagenome sequencing: secondary peat swamp forest (two top-layer samples and two bottom-layer samples), land preparation (three top-layer samples and three bottom-layer samples), and oil palm plantation (two top-layer samples and two bottom-layer samples; Table S3; *n* = 14).

### Metagenomic sequencing and analyses

The DNA was quantified with NanoPhotometer P360 (Implen GmbH, Germany) and QubitTM 4 Fluorometer (Invitrogen, Singapore) prior to metagenomic sequencing. Metagenomic library preparation and shotgun sequencing were performed in NovogeneAIT Genomics (Singapore). Briefly, total DNA was randomly sheared into fragments. The fragments were end-repaired, polyadenylated, and ligated with Illumina adapters before PCR amplification. Quantified libraries were sequenced using the NovaSeq 6000 platform (Illumina, CA, USA) with 2 × 150 paired-end read chemistry to a sequencing depth of 40 Gbp. The raw paired-end reads from the 14 metagenomes were processed with BBTools v38.94. The data set coverage was estimated with Nonpareil v3.304 (88, 89). Information on the sequencing effort and estimated average coverage is described in Table S3; Fig. S2.

The read-based classifications were performed with Kraken2 v2.1.2 mapped to a non-redundant NCBI *nt* database (90). The relative abundance of microbiome profiles was then re-estimated with Bracken v2.6.2 and converted to *biom* files using kraken-biom v1.0.1 for further analyses (91, 92).

For gene-based analysis, the clean reads were error-corrected with *bbcms.sh* (BBTools) and assembled with MEGAHIT v1.2.9 (89, 93). Assembled contigs were then assessed with metaQUAST v5.0.2 mapped with *bbmap.sh* (BBTools) and predicted for protein-coding sequences (CDSs) using Prodigal v2.6.3 (89, 94–96). Functional annotation was performed using eggNOG-mapper v2.1.7 with eggNOG database v5.0.2 and DIAMOND in *--iteration* mode (97–99). The annotation best hits were screened for genes acting on CH_4_ and nitrogen transformation processes with core genes listed in Tables S14 and S15 based on gene names and KEGG (Kyoto Encyclopedia of Genes and Genomes) Orthology entries. Carbohydrate-active enZymes (CAZy) were identified through the CAZy database using dbCAN v3.0.7 with HMMER and DIAMOND (99–101). Putative CDSs with at least one positive hit were selected for further annotation with NCBI non-redundant *nr* database, Swiss-Prot-curated protein sequence database, and Protein Data Bank database (*pdbaa*) using DIAMOND v2.1.8.162 and *blastx* (99). Distant homologs identified as false positives were removed.

In addition, the “.daa” outputs from DIAMOND alignments to the *nr* database were used to assign taxonomic classification through the MEGANIZER program based on the naïve lowest common ancestor algorithm in MEGAN6 (99, 102, 103). In addition, assembled contigs were binned with CONCOCT v1.0.0, metaBAT2 v2.12.1, and MaxBin2 v2.2.6 with default parameters and consolidated within metaWRAP v.1.3.2 to recover MAGs (104–107). Bin quality was determined with CheckM2 v1.0.2, and draft bins with more than 50% completeness and less than 10% contamination based on MIMAG were checked with MAGPurify v2.1.2 to remove incorrectly binned contigs (108–110). The draft MAGs were dereplicated with dRep v3.0.0 with MAGs passing the quality threshold taxonomically classified by the Genome Taxonomy Database toolkit (GTDB-Tk v2.1.1, R207 v2) and functionally annotated with Distilled and Refined Annotation of Metabolism (DRAM v1.4.6) (111–113). The detailed parameters used in the metagenomic analyses are available in the Supplementary Information S3.

### Data analyses

Statistical analyses and visualizations were conducted in R v4.3.1 with RStudio v.2023.03.1 (114, 115). The R packages used were *stats* v4.3.1, *phyloseq* v1.44.0, and *vegan* v2.6–4 (115–117). Alpha diversity was estimated using *Nonpareil* sequence diversity (*N_d_*) based on rarefied coverage that combined richness and evenness to represent total diversity. The *Nonpareil* sequence diversity correlates with classic diversity indexes. Beta-diversity was analyzed with non-metric multidimensional scaling based on Bray-Curtis distances computed using the *metaMDS* function in the *vegan* package. The bi-plot was constructed with the *envfit* function to plot peat chemical properties and GHG measurements to the prokaryotic communities.

The relative abundance of selected functional genes was quantified by mapping metagenomic reads to all predicted sequences, which were normalized with gene length to represent gene abundance within the microbial communities. Heatmaps for selected soil respiration genes, CAZymes, and COGs were constructed based on *Z*‐score transformed data to improve normality and homogeneity of variances. Mantel test with Bray-Curtis distances using Spearman’s rank correlation was used to determine environmental variables and soil greenhouse gases correlation with microbial community composition and genes related to production and consumption of CH_4_ and N_2_O. The ratio of *mcrA* to *pmoA* indicated the ratio between methanogenesis and CH_4_ oxidation. Nitrification was compared to denitrification through the *amoA* to the sum of *nirK* and *nirS* ratio. The sum of *nirK* and *nirS* genes to *nosZ* and the ratio of *norB* to *nosZ* were used as indicators of denitrification-driven gaseous nitrogen loss potential. Data visualization was performed with *ggplot2* v3.4.2, *ComplexHeatmap* v2.16.0, *pheatmap* v1.0.12, *cowplot* v1.1.1, and *patchwork* v1.1.2 R packages (118–120). Additional information for the data analyses can be found in the Supplementary Information S4.

## Data Availability

Sequence data are available through NCBI Sequence Read Archive (SRA) under BioProject accession number PRJNA937402. The present study did not generate codes, and the tools used in the data analysis applied default parameters unless specified otherwise.

## SUPPLEMENTAL MATERIAL

Supplemental material aem.01986-24-s0001.docx Supplemental methods, Tables S1 to S15, and Figures S1 to S7.

ASM does not own the copyrights to Supplemental Material that may be linked to, or accessed through, an article. The authors have granted ASM a non-exclusive, world-wide license to publish the Supplemental Material files. Please contact the corresponding author directly for reuse.
