# Supplementary material for: Temporal dynamics of soil microbial C and N cycles with GHG fluxes in the transition from tropical peatland forest to oil palm plantation
Source: Appl Environ Microbiol. 2024 Dec 23;91(1):e01986-24. doi: 10.1128/aem.01986-24 (PMC11784229; doi:10.1128/aem.01986-24)
Supplement: Supplemental material — Supplemental methods, Tables S1 to S15, and Figures S1 to S7. [file aem.01986-24-s0001.docx]

**Supplementary Information**

Temporal dynamics of soil microbial C and N cycles with GHG fluxes in the transition from tropical peatland forest to oil palm

Frazer Midot^1,2*^, Kian Mau Goh^2*^, Kok Jun Liew^2^, Sharon Yu Ling Lau^1^, Mikk Espenberg^3^, Ülo Mander^3^, Lulie Melling^1^

*^1^Sarawak Tropical Peat Research Institute, 94300 Kota Samarahan, Sarawak, Malaysia.*

*^2^Faculty of Science, Universiti Teknologi Malaysia, 81310, Skudai, Johor, Malaysia.*

*^3^Institute of Ecology and Earth Sciences, University of Tartu, Tartu, Estonia*

*Corresponding authors: Frazer Midot (frazer.m@sarawak.gov.my), Kian Mau Goh (gohkianmau@utm.my)

# **Supplementary Information S1: Peat chemical analysis**

Peat pH was measured using a pH meter (Metrohm 827, Switzerland) of a 2:5 (w/v) peat suspension in ultrapure water. The total carbon and nitrogen content in peat samples was measured with a CN analyzer (Leco TruMac CN, USA). Peat humification was determined using the pyrophosphate soluble index (PSI) by measuring 550 nm absorbance of an 18-hour extract that was filtered through a Whatman Filter Paper No.5 from 0.5 g soil in 50 ml sodium pyrophosphate (0.025 M) (Perkin Elmer UV/Vis Lambda 35, USA). Ammonium (NH_4_^+^), nitrate (NO_3_^-^) and phosphate (PO_4_^-3^) concentrations were determined with an ion chromatograph (Metrohm 716 Compact IC, Switzerland) by measuring ion concentration in a peat extract prepared from 6.0 g peat suspension in ultrapure water shaken for an hour.

# **Supplementary Information S2: Soil respiration measurements**

At each sampling point, 250 ml of air was collected for CO_2_ gas sampling using a 500 ml Tedler® bag and syringe. Air samples for CH_4_ and N_2_O were collected (20 ml) using syringes and transferred to 15 ml pre-vacuumed GC gas vials. After the chamber was removed, the groundwater level was measured through auger holes (n = 8).

The CO_2_ gas samples were collected in 250 ml Tedler bags and analyzed *on-site* (within six hours) using Infrared CO_2_ gas analyses (Fuji Electric ZFPGC11, Japan). For CH_4_ and N_2_O, the 15 ml gas vials were transported back to the laboratory. The CH_4_ gas concentration was measured using a gas chromatography system with a flame ionization detector (FID) (Agilent 7890A, USA). The N_2_O concentration was determined using a gas chromatography system with an electron capture detector (Agilent 7890A, USA).

# **Supplementary Information S3: Metagenomic data processing**

With BBTools, Illumina adapters and common read contaminants were trimmed from the raw paired-end sequence reads. Reads were then quality-trimmed and filtered with *bbduk*.*sh* using the following parameters: *trimpolyg=5 trimq=10 maxns=4 maq=3 minlen=51 minlenfraction=0.33*. Trimmed reads were masked against human, cat, dog, and mouse reference genomes available through BBTools. The clean reads were produced with *bbmap.sh,* using a minimum alignment identity of 93% (*minid=0.93*) (1).

The clean reads were then assessed for coverage using a statistical program called Nonpareil v3.304 to estimate the coverage from sequencing effort using read redundancy (2, 3). The *npo* files were plotted as Nonpareil curves with the *Nonpareil.set* function in the Nonpareil R package.

The clean reads were classified using Kraken2 v2.1.2, a *k-mer* matching algorithm (4). The reference database (Nov 2021) was extracted from the NCBI non-redundant nucleotide database to classify the paired-end reads. After Kraken2 classification, the relative abundances of microbes in the samples were re-estimated using Bracken v2.6.2 with the Bracken database file generated from a read length of 150 and a k-mer length of 35 (5). Bracken report files were combined and converted into *biom* files using kraken-biom v1.0.1 (6).

Clean reads were error corrected using *bbcms.sh* from BBTools v38.94, a scalable error correction tool for metagenomic datasets with *k=31 hashes=3 mincount=2 hcf=0.4 bits=4 ecc=t merge=t* (1). Next, error-corrected datasets were assembled with MEGAHIT v1.2.9 using -*-presets meta-large* that specify minimum k-mer size 27 (*--k-min 27*) and maximum k-mer 127 (*--k-max 27*) with an increment of 10 k-mer size for each step (*--k-step 10*) (7). Contigs smaller than 200 bp were removed with *--min-contig-len 200*.

The quality of the generated assemblies from MEGAHIT was evaluated with metaQUAST 5.0.2 (8, 9). The metaQUAST program calculates basic assembly statistics based on contigs with sizes above 1,000 bp. Calculated statistics are the total number of contigs, the number of contigs above various lengths (1 kbp, 5 kbp, 10 kbp, 25 kbp and 50 kbp), the total length of contigs above various lengths (1 kbp, 5 kbp, 10 kbp, 25 kbp and 50 kbp), N50 length and L50 length. Cleaned reads were also mapped to filtered contigs with *bbmap.sh* (BBTools v38.94) with *ambiguous* *= random*.

The assembled contigs were processed for predicted protein-coding sequences using Prodigal v2.6.3 in *meta* mode and the *-m* argument to treat the stretch of “N” as masked sequences and would not predict any genes across these sequences (10). The protein-coding sequences under 75 nucleotides or 25 amino acids were discarded with seqkit v2.1.0 (11). The protein-coding sequences that passed the threshold were annotated using eggNOG-mapper v2.1.7 with the eggNOG database v5.0.2 for inferring orthology (12, 13). The protein search with eggNOG-mapper was done with Double Index Alignment of Next-Generation sequencing Data (DIAMOND) iterative mode combined with *--sensmode more-sensitive* and 0.001 e-value cut-offs (14). The best seed orthologs are channeled into orthology assignments and annotated against the eggNOG annotation database.

Selected genes for mining microbial methane production and consumption are listed in Table S14. Selected genes in the nitrogen cycle are nitrification, denitrification, dissimilatory nitrate reduction to ammonia and nitrogen fixation process, which are listed in Table S15. All protein-coding sequences were annotated to the Carbohydrate-Active enZymes (CAZymes) database through run_dbcan v3.0.7 using HMMER and DIAMOND.

Sequences with positive hits for selected genes and CAZymes were extracted using exonerate v2.4.0 through *fastaindex* and *fastafetch* options for further annotation. The extracted FASTA format sequences were further annotated with NCBI non-redundant *nr* database, Uniprot curated protein sequence database (Swiss-Prot) and Protein Data Bank database (pdbaa) using DIAMOND v2.1.8.162 with *blastx --evalue 1e-05 --max-target-seqs 3 --max-hsps 3* that include information on sequence homologs and closest taxonomic annotation. False positives were manually removed. Putative proteins with at least a hit in two protein databases and a minimal threshold of 20% identity were retained.

To recover metagenome-assembled genomes (MAGs), the contig assemblies were initially binned using CONCOCT (v1.0.0), MaxBin2 (v2.2.6) and metaBAT2 (v2.12.1) within the binning module of metaWRAP (v1.3.2) with the following parameters: minimum contig length of 1,000 bp (-l 1000) and usage of universal marker genes for binning (--universal) (15-18). All bins recovered were refined, reassembled and consolidated using the metaWRAP’s “bin_refinement” and “reassemble_bins” module to a cut-off genome bins completeness of above 50% (-c 50) and contamination under 10% (-x 10). These bins were further refined using MAGPurify v2.1.2 to remove contamination or incorrectly binned contigs (19). Positional arguments used in MAGpurify were “phylo-markers”, “clade-markers”, “tetra-freq”, “gc-content”, “known-contam”, and “clean-bin”. Based on MIMAG, for MAG to be determined as “high-quality”, the genome should be more than 90% complete and less than 5% contaminated (20). The “medium-quality” MAGs have genomes with equal or more than 50% estimated genome completion and less than 10% contamination level (20). With default parameters, draft MAGs were dereplicated with dRep before taxonomic classification with Genome taxonomy database toolkit (GTDB-Tk v2.1.1, R207 v2) and functional annotation with Distilled and Refined Annotation of Metabolism (DRAM v1.4.6) (21, 22).

# **Supplementary Information S4: Data analyses**

The chemical properties expressed in percentage were transformed with arcsine square root transformations before subsequent analyses. One-way analysis of variance (ANOVA), followed by Tukey’s honestly significance difference (HSD) in the R *stats* v4.3.1, was used to analyze the differences in peat chemical properties and GHG measurements in different land uses, depths, and seasons.

For taxonomic analyses, the combined *biom* files from Kraken2 annotations were imported into the *phyloseq* v1.44.0 R package for further downstream analyses (23, 24). Prokaryotes’ relative abundance was analyzed with the Kruskal-Wallis test, followed by the pairwise Wilcoxon test for *post* *hoc* examination to assess the effect of different land use, seasons, and depth towards microbial communities.

Alpha diversity of the tropical peatland microbiome datasets was estimated using the Nonpareil diversity index (2). Beta diversity was assessed using non-metric multidimensional scaling (NMDS) based on Bray-Curtis distances to characterize the microbial profiles in different land uses. The NMDS was performed using the *metaMDS* function in the vegan v2.6.4 R package (25). The *envfit* function in the vegan R package was used to plot the peat chemical properties and GHGs in the NMDS ordination plot. The statistically significant difference was set at *p<*0.05.

Dispersion homogeneity was assessed with betadisper to determine that observed significant differences were due to the tested factors and not the within-group variabilities. The permutational multivariate analysis of variance (PERMANOVA) implemented in the adonis2 function in vegan was used to assess the effect of land-use change, seasonal variation and sampling depth with 9,999 times permutations with a significance value of 0.05.

The distribution and cluster analyses of Clusters of Orthologous Genes (COG) categories, KEGG Orthology (KO) entries with genes related to methane production, methane oxidation, ammonia oxidation, denitrification, DNRA and nitrogen fixation, including CAZymes throughout different land use were illustrated with heatmaps generated through *ComplexHeatmap* v2.16.0 with *clustering_distance_cols = correlation* (26).

# **Table S1** Soil greenhouse gases (GHG) relative humidity, air and soil temperature, including groundwater table values during the study period. Values are mean ± SD, followed by different lowercase alphabets to indicate significant differences at p<0.05. Significant values (*p*<0.05) are in bold font and marked with an asterisk (*). SD = Standard Deviation

| **Land use** | **Secondary**  **Peat Swamp Forest** | | **Land Preparation** | | | **Oil Palm**  **Plantation** | | **one-way ANOVA** | |
| --- | --- | --- | --- | --- | --- | --- | --- | --- | --- |
| **Sample ID** | **Jan-**  **2016** | **Aug-2016** | **Aug-2017** | **Jan-**  **2018** | **Mar-**  **2018** | **Jan-**  **2020** | **Aug-2020** | **Main Effect** | |
| **Season** | **Wet** | **Dry** | **Dry** | **Wet** | **Wet** | **Wet** | **Dry** | **Land use** | **Season** |
| **Water table**  **(cm)** | 8.5 ±  2.2a | -11.1 ±  2.7b | -104.0 ±  2.1c | -100.9 ±  6.8d | -87.9 ±  2.8e | -52.8 ±  5.5f | -50.7 ±  4.1g | **<0.001*** | **<0.001*** |
| **Soil moisture content (%)** | 89.7 ±  6.7d | 72.2 ±  11.1ab | 59.5 ±  15.8bc | 84.9 ±  5.8a | 57.6 ±  11.4c | 59.7 ±  16.7bc | 51.8 ±  8.9c | **0.025*** | **0.327** |
| **Soil temperature (°)** | 28.0 ±  0.3b | 26.6 ±  0.3c | 28.1 ±  1.0b | 27.2 ±  0.4c | 28.8 ±  0.4ab | 29.0 ±  0.4a | 28.7 ±  0.7a | **<0.001*** | **0.067** |
| **Air temperature (°)** | 26.9 ±  0.7d | 29.6 ±  0.3c | 30.7 ±  3.3c | 26.4 ±  1.0d | 36.4 ±  1.0a | 33.0 ±  2.2ab | 33.8 ±  1.2b | **<0.001*** | **0.573** |
| **Relative humidity (%)** | 96.5 ±  3.5a | 82.8 ±  3.2b | 80.3 ±  16.3bc | 77.7 ±  6.0b | 60.1 ±  8.2d | 71.5 ±  8.3cd | 69.0 ±  3.7cd | **<0.001*** | **0.555** |
| **CO_2_**  **(****mg C m^-2^ h^-1^)** | 163.5 ±  39.6b | 267.2 ±  78.9b | 242.2 ±  46.2b | 318.2 ±  61.0b | 294.2 ±  48.2b | 618.6 ±  102.8a | 220.5 ±  41.2b | **0.008*** | **0.020*** |
| **CH_4_**  **(****µg C m^-2^ h^-1^)** | 335.9 ±  179.0a | 55.7 ±  60.4a | 77.0 ±  54.9a | 224.2 ±  160.6a | -66.6 ±  39.9a | 62.1 ±  136.5a | -64.5 ±  68.9a | 0.263 | 0.178 |
| **N_2_O**  **(****µg N m^-2^ h^-1^)** | -11.2 ±  14.5b | -6.0 ±  17.4b | 66.2 ±  40.2b | 51.3 ±  22.8b | 338.3 ±  40.0b | 2551.3 ±  457.9a | 468.6 ±  233.8b | **<0.001*** | **<0001*** |

# **Table S2** Tropical peat chemical properties one-way ANOVA. Significant values (p<0.05) are in bold font and marked with an asterisk (*).

| **Peat Chemical Properties** | **one-way ANOVA** | | |
| --- | --- | --- | --- |
|  | **Main Effect** | | |
|  | **Land use** | **Season** | **Depth** |
| **pH** | 0.255 | 0.241 | 0.090 |
| **Pyrophosphate Soluble Index (%)** | **<0.001*** | 0.521 | **0.001*** |
| **Total Carbon (%)** | **0.007*** | 0.724 | **<0.001*** |
| **Total Nitrogen (%)** | 0.334 | 0.494 | **0.003*** |
| **C:N Ratio** | 0.180 | 0.411 | **<0.001*** |
| **Nitrate (ppm)** | **0.045*** | 0.129 | 0.650 |
| **Ammonium (ppm)** | **0.004*** | 0.219 | **<0.001*** |
| **Phosphate (ppm)** | **<0.001*** | 0.490 | **<0.001*** |

# **Table S3** Summary of sequencing data characteristics. Sample ID is Month_Year_Depth; Land preparation = LP; Oil Palm Plantation = OPP.

| **Sample ID** | **Land-use** | **Season** | **Depth**  **(cm)** | **NCBI BioProject** | **NCBI Sample Accession** | **Read Length** | **Total bases (Gbp)** | **Raw Reads**  **(10^8^)** | **Clean Reads (10^8^)** |
| --- | --- | --- | --- | --- | --- | --- | --- | --- | --- |
| **Jan-2016_T** | Forest | Wet | 0-25 | PRJNA937402 | SAMN33440578 | 151 | 21.3 | 1.423 | 1.422 |
| **Jan-2016_B** | Forest | Wet | 25-50 | PRJNA937402 | SAMN33440579 | 151 | 24.0 | 1.601 | 1.600 |
| **Aug-2016_T** | Forest | Dry | 0-25 | PRJNA937402 | SAMN33440580 | 151 | 40.8 | 2.723 | 2.720 |
| **Aug-2016_B** | Forest | Dry | 25-50 | PRJNA937402 | SAMN33440581 | 151 | 44.5 | 2.966 | 2.963 |
| **Aug-2017_T** | LP | Dry | 0-25 | PRJNA937402 | SAMN33440582 | 151 | 40.9 | 2.724 | 2.721 |
| **Aug-2017_B** | LP | Dry | 25-50 | PRJNA937402 | SAMN33440583 | 151 | 42.8 | 2.857 | 2.853 |
| **Jan-2018 _T** | LP | Wet | 0-25 | PRJNA937402 | SAMN33440584 | 151 | 46.6 | 3.106 | 3.101 |
| **Jan-2018_B** | LP | Wet | 25-50 | PRJNA937402 | SAMN33440585 | 151 | 42.6 | 2.840 | 2.837 |
| **Mar-2018_T** | LP | Wet | 0-25 | PRJNA937402 | SAMN33440586 | 151 | 47.4 | 3.157 | 3.153 |
| **Mar-2018_B** | LP | Wet | 25-50 | PRJNA937402 | SAMN33440587 | 151 | 42.0 | 2.797 | 2.794 |
| **Jan-2020_T** | OPP | Wet | 0-25 | PRJNA937402 | SAMN33440588 | 151 | 44.0 | 2.931 | 2.927 |
| **Jan-2020_B** | OPP | Wet | 25-50 | PRJNA937402 | SAMN33440589 | 151 | 41.8 | 2.785 | 2.782 |
| **Aug-2020_T** | OPP | Dry | 0-25 | PRJNA937402 | SAMN33440590 | 151 | 40.0 | 2.664 | 2.662 |
| **Aug-2020_B** | OPP | Dry | 25-50 | PRJNA937402 | SAMN33440591 | 151 | 42.3 | 2.821 | 2.818 |

# **Table S4** Classified metagenomic sequences and relative abundance of domain-level taxa for tropical peat samples.

| **Sample ID** | **Classified Paired Reads (10^8^)** | **Domain Relative Abundance** | | | |
| --- | --- | --- | --- | --- | --- |
|  |  | **Archaea**  **(%)** | **Bacteria**  **(%)** | **Eukaryota**  **(%)** | **Viruses**  **(%)** |
| **Jan-2016_T** | 0.242 | 0.9 | 78.6 | 20.1 | 0.4 |
| **Jan-2016_B** | 0.244 | 1.5 | 75.1 | 23.1 | 0.4 |
| **Aug-2016_T** | 0.431 | 0.9 | 76.4 | 22.3 | 0.4 |
| **Aug-2016_B** | 0.393 | 1.2 | 69.8 | 28.5 | 0.5 |
| **Aug-2017_T** | 0.438 | 1.0 | 76.4 | 22.2 | 0.4 |
| **Aug-2017_B** | 0.388 | 1.9 | 69.4 | 28.3 | 0.4 |
| **Jan-2018_T** | 0.477 | 1.7 | 73.8 | 24.0 | 0.4 |
| **Jan-2018_B** | 0.403 | 1.7 | 71.6 | 26.3 | 0.4 |
| **Mar-2018_T** | 0.486 | 1.7 | 74.4 | 23.5 | 0.4 |
| **Mar-2018_B** | 0.411 | 2.2 | 72.4 | 25.0 | 0.4 |
| **Jan-2020_T** | 0.467 | 2.9 | 73.5 | 23.2 | 0.4 |
| **Jan-2020_B** | 0.429 | 2.8 | 72.7 | 24.0 | 0.4 |
| **Aug-2020_T** | 0.387 | 3.1 | 70.4 | 26.1 | 0.4 |
| **Aug-2020_B** | 0.408 | 3.0 | 70.9 | 25.7 | 0.4 |

# **Table S5** Summary statistics for Nonpareil analysis based on *kmer* diversity of forward reads. The average sequencing coverage was determined using the Nonpareil curve. Metagenomic dataset coverage estimates varied across land use types, with secondary forest samples ranging from 64% to 67%, land preparation samples from 64% to 73.8% and plantation samples exhibiting the highest coverage at 78% to 84%.

| **Sample ID** | **Estimated Average Coverage (%)** | **Nonpareil Sequence-Diversity (N_d_)** |
| --- | --- | --- |
| **Jan-2016_B** | 65.1 | 22.330 |
| **Jan-2016_T** | 64.8 | 22.173 |
| **Aug-2016_B** | 67.3 | 22.552 |
| **Aug-2016_T** | 63.9 | 22.814 |
| **Aug-2017_B** | 69.4 | 22.451 |
| **Aug-2017_T** | 64.0 | 22.820 |
| **Jan-2018_B** | 72.8 | 22.084 |
| **Jan-2018_T** | 73.8 | 22.044 |
| **Mac-2018_B** | 68.0 | 22.467 |
| **Mac-2018_T** | 67.5 | 22.696 |
| **Jan-2020_B** | 83.8 | 20.925 |
| **Jan-2020_T** | 83.9 | 20.676 |
| **Aug-2020_B** | 81.2 | 21.204 |
| **Aug-2020_T** | 78.1 | 21.188 |
| **Average** | 71.7 | 22.030 |
| **Standard deviation** | 7.315 | 0.728 |

# **Table S6** Kruskal-Wallis test on the relative abundance of tropical peatland prokaryotes. Significant values (p<0.05) are in bold and marked with an asterisk (*).

| **Phylum** | **Kruskal-Wallis test** | | |
| --- | --- | --- | --- |
|  | ***p*-value** | | |
| **Domain Bacteria** | **Land use** | **Season** | **Depth** |
| *Proteobacteria* | 0.644 | 0.796 | **0.006*** |
| *Actinobacteria* | 0.443 | 0.606 | **0.006*** |
| *Acidobacteria* | 0.598 | 0.156 | 0.064 |
| *Planctomycetes* | 0.172 | **0.014*** | 0.749 |
| *Firmicutes* | 0.696 | 0.302 | **0.013*** |
| *Bacteroidetes* | 0.497 | 0.137 | **0.021*** |
| Candidatus *Cloacimonetes* | 0.428 | 0.121 | 0.064 |
| *Cyanobacteria* | 0.524 | 0.155 | **0.030*** |
| *Chloroflexi* | 0.651 | 0.245 | **0.025*** |
| *Verrucomicrobia* | **0.012*** | 0.519 | 0.180 |
| **Domain Archaea** | | | |
| *Euryarchaeota* | **0.006*** | 0.519 | 0.749 |
| “*Candidatus* Thermoplasmatota*”* | **0.006*** | 0.796 | 0.482 |
| *Thaumarchaeota* | **0.038*** | 0.699 | 0.338 |
| “*Candidatus* Korarchaeota” | 0.368 | 0.796 | **0.004*** |

# **Table S****7** Permutation test for homogeneity of multivariate dispersions using *betadisper* in the *vegan* package at 9,999 permutations.

| ***betadisper*** | **F value** | ***P* value** |
| --- | --- | --- |
| **Land use** | 0.635 | 0.548 |
| **Depth** | 0.264 | 0.617 |
| **Season** | 0.405 | 0.527 |

#

# **Table S8** PERMANOVA analysis of classified prokaryotes (9,999 permutations) comparing land use, depth and season. Significant values (*P*<0.05) are in bold and marked with an asterisk (*).

| **PERMANOVA** | **R^2^** | **F** | ***P* value** |
| --- | --- | --- | --- |
| **Land use** | 0.5111 | 10.3883 | **0.0001*** |
| **Depth** | 0.2188 | 8.8940 | **0.0002*** |
| **Season** | 0.0486 | 1.9753 | 0.1393 |
| **Residual** | 0.2214 | - | - |
| **Total** | 1.0000 | - | - |

# **Table S9** Peat properties and GHG emissions correlation to prokaryote diversity. Significant values (*p*<0.05) are in bold and marked with an asterisk (*).

| **Variables** | **Prokaryote Diversity** | |
| --- | --- | --- |
|  | ***r*** | ***p*** |
| **Groundwater level** | 0.288 | **0.013*** |
| **Soil moisture content (%)** | 0.311 | **0.012*** |
| **Soil temperature (°C)** | 0.3395 | **0.071*** |
| **pH** | 0.214 | **0.044*** |
| **PSI** | 0.559 | **<0.001*** |
| **Total Carbon** | 0.063 | 0.300 |
| **Total Nitrogen** | 0.107 | 0.152 |
| **C:N Ratio** | 0.250 | **0.025*** |
| **Ammonium** | 0.315 | **0.009*** |
| **Nitrate** | 0.106 | 0.200 |
| **Phosphate** | 0.206 | **0.047*** |
| **CO_2_ (mg C m^−2^ h^−1^)** | 0.115 | 0.185 |
| **CH_4_ (µg C m^−2^ h^−1^)** | 0.149 | 0.096 |
| **N_2_O (µg N m^−2^ h^−1^)** | 0.378 | **0.002*** |

# **Table S10** Assembly statistics of tropical peat contigs.

| **Sample** | **GC (%)** | **Total Length (Mbp)** | **Total Contigs (10^6^)** | **Largest Contig  (Mbp)** | **Number of Contigs** | | | | | **N50** | **L50** |
| --- | --- | --- | --- | --- | --- | --- | --- | --- | --- | --- | --- |
|  |  |  |  |  | **≥1,000 bp** | **≥5,000 bp** | **≥10,000 bp** | **≥25,000 bp** | **≥50,000 bp** |  |  |
| **Jan-2016_T** | 63.0 | 1017 | 0.964 | 0.164 | 272,150 | 12,131 | 2,874 | 366 | 35 | 1,089 | 231,468 |
| **Jan-2016_B** | 60.3 | 1103 | 1.083 | 0.190 | 301,604 | 9,868 | 2,177 | 297 | 52 | 1,044 | 277,539 |
| **Aug-2016_T** | 61.9 | 2090 | 1.970 | 0.136 | 560,966 | 24,803 | 6,215 | 908 | 114 | 1,098 | 470,398 |
| **Aug-2016_B** | 59.9 | 2229 | 2.007 | 0.211 | 589,908 | 31,674 | 8,324 | 1,237 | 227 | 1,173 | 442,540 |
| **Aug-2017_T** | 62.3 | 2075 | 1.983 | 0.248 | 566,267 | 21,739 | 5,013 | 740 | 126 | 1,085 | 485,785 |
| **Aug-2017_B** | 59.1 | 2135 | 1.928 | 0.239 | 553,168 | 29,862 | 8,703 | 1,535 | 356 | 1,160 | 421,208 |
| **Jan-2018_T** | 62.2 | 2.130 | 1.848 | 0.277 | 558,730 | 32,988 | 9,477 | 1,641 | 338 | 1,245 | 382,558 |
| **Jan-2018_B** | 60.5 | 2.005 | 1.760 | 0.231 | 528,109 | 31,192 | 8,417 | 1,279 | 262 | 1,224 | 371,361 |
| **Mar-2018_T** | 62.3 | 2.268 | 2.148 | 0.191 | 614,065 | 24,375 | 6,344 | 1,055 | 188 | 1,096 | 517,579 |
| **Mar-2018_B** | 61.5 | 1.980 | 1.835 | 0.157 | 516,847 | 25,945 | 6,993 | 1,076 | 197 | 1,120 | 418,209 |
| **Jan-2020_T** | 64.1 | 1.641 | 1.238 | 0.412 | 405,120 | 33,731 | 11,908 | 2,803 | 777 | 1,576 | 195,859 |
| **Jan-2020_B** | 62.9 | 1.788 | 1.283 | 0.444 | 435,274 | 41,017 | 14,821 | 3,348 | 823 | 1,753 | 186,324 |
| **Aug-2020_T** | 63.1 | 1.708 | 1.328 | 0.375 | 433,973 | 33,148 | 10,937 | 2,375 | 621 | 1,501 | 226,771 |
| **Aug-2020_B** | 62.7 | 1.864 | 1.386 | 0.465 | 475,488 | 38,986 | 13,287 | 2,757 | 685 | 1,633 | 223,482 |

# **Table S11** Mantel test of peat properties and core genes in methanogenesis (*mcrABC*) and methanotrophy (*pmoABC*, *mmoX*). Significant values (*p*<0.05) are in bold and marked with an asterisk (*).

| **Variables** | ***mcrABC*** | | ***pmoABC*** | | ***mmoX*** | |
| --- | --- | --- | --- | --- | --- | --- |
|  | ***r*** | ***p*** | ***r*** | ***p*** | ***r*** | ***p*** |
| **Groundwater level** | 0.155 | 0.115 | 0.373 | **0.005*** | 0.065 | 0.231 |
| **pH** | -0.136 | 0.843 | -0.143 | 0.866 | 0.058 | 0.254 |
| **Soil moisture content (%)** | 0.148 | 0.132 | 0.340 | **0.012*** | 0.256 | **0.036*** |
| **Soil temperature (°C)** | -0.083 | 0.705 | -0.076 | 0.684 | 0.327 | **0.017*** |
| **PSI** | 0.283 | **0.022*** | 0.215 | 0.058 | 0.186 | 0.065 |
| **Total Carbon** | -0.115 | 0.698 | -0.244 | 0.941 | 0.003 | 0.426 |
| **Total Nitrogen** | 0.153 | 0.145 | -0.178 | 0.914 | 0.561 | **0.002*** |
| **CN Ratio** | 0.243 | **0.030*** | -0.085 | 0.755 | 0.548 | **0.002*** |
| **Ammonium** | 0.087 | 0.253 | 0.006 | 0.426 | 0.462 | **0.008*** |
| **Nitrate** | -0.168 | 0.809 | -0.189 | 0.875 | -0.073 | 0.641 |
| **Phosphate** | 0.195 | 0.107 | -0.063 | 0.639 | 0.409 | **0.010*** |
| **CO_2_ (mg C m^-2^ h^-1^)** | 0.386 | **0.031*** | 0.132 | 0.226 | -0.012 | 0.467 |
| **CH_4_ (µg C m^-2^ h^-1^)** | 0.159 | 0.129 | 0.354 | **0.010*** | 0.189 | 0.733 |
| **N_2_O (µg N m^-2^ h^-1^)** | 0.218 | 0.118 | -0.071 | 0.586 | 0.074 | 0.229 |

# **Table S12** Mantel test of peat properties and core genes in relation to N_2_O production and consumption (*nosZ*). Significant values (*p*<0.05) are in bold and marked with an asterisk (*).

| **Variables** | ***amoABC*** | | ***nirK+nirS*** | | ***norB*** | | ***nosZ*** | |
| --- | --- | --- | --- | --- | --- | --- | --- | --- |
|  | ***r*** | ***p*** | ***r*** | ***p*** | ***r*** | ***p*** | ***r*** | ***p*** |
| **Groundwater level** | 0.373 | **0.005*** | 0.227 | **0.040*** | 0.093 | 0.217 | 0.136 | 0.134 |
| **Soil moisture content (%)** | 0.340 | **0.012*** | 0.120 | 0.155 | 0.015 | 0.414 | 0.194 | 0.078 |
| **Soil temperature (°C)** | -0.076 | 0.690 | 0.187 | 0.075 | -0.135 | 0.875 | 0.0662 | 0.265 |
| **pH** | -0.143 | 0.866 | –0.052 | 0.599 | –0.062 | 0.650 | -0.228 | 0.998 |
| **PSI** | 0.215 | 0.058 | 0.156 | 0.098 | 0.145 | 0.134 | -0.121 | 0.829 |
| **Total Carbon** | -0.244 | 0.941 | –0.147 | 0.823 | ­–0.208 | 0.891 | -0.164 | 0.807 |
| **Total Nitrogen** | -0.178 | 0.914 | ­–0.108 | 0.780 | 0.297 | **0.019*** | -0.163 | 0.893 |
| **CN Ratio** | -0.085 | 0.755 | –0.048 | 0.634 | 0.343 | **0.004*** | -0.126 | 0.857 |
| **Ammonium** | 0.006 | 0.426 | –0.00043 | 0.431 | –0.003 | 0.461 | -0.158 | 0.905 |
| **Nitrate** | -0.189 | 0.875 | 0.005 | 0.387 | –0.014 | 0.459 | -0.272 | 0.9995 |
| **Phosphate** | -0.063 | 0.639 | 0.053 | 0.325 | ­–0.014 | 0.499 | -0.161 | 0.894 |
| **CO_2_ (mg C m^-2^ h^-1^)** | 0.132 | 0.226 | 0.342 | **0.023*** | 0.271 | 0.064 | 0.066 | 0.317 |
| **CH_4_ (µg C m^-2^ h^-1^)** | 0.354 | **0.010*** | 0.036 | 0.343 | 0.414 | 0.349 | 0.214 | 0.066 |
| **N_2_O (µg N m^-2^ h^-1^)** | -0.071 | 0.586 | 0.411 | **0.005*** | 0.125 | 0.235 | -0.102 | 0.696 |

# **Table S13** Differential analysis of dominant taxa related to CH_4_ and N_2_O transformation genes compared to secondary peat swamp forest.

| **Pathway** | **Core genes** | **Taxa^#^** | **Land Preparation** | **Oil Palm Plantation** |
| --- | --- | --- | --- | --- |
| **Methanogenesis** | *mcrA* | *Methanocellales* (*Euryarchaeota*, *Methanomicrobia*)  *Methanosarcinales* (*Euryarchaeota*, *Methanomicrobia*) | −  + | +  + |
| **Methane oxidation** | *pmoA* | *Methylocystis* (*Alphaproteobacteria*, *Hyphomicrobiales*)  *Methylosinus* (*Alphaproteobacteria*, *Hyphomicrobiales*)  *Bradyrhizobium* (*Alphaproteobacteria*, *Hyphomicrobiales*) | −  −  − | −  −  − |
| **Nitrogen fixation** | *nifH* | *Hyphomicrobiales* (*Alphaproteobacteria*)  *Rhodospirilales* (*Alphaproteobacteria*)  *Burkholderiales* (*Betaproteobacteria*)  *Desulfobacterales* (*Deltaproteobacteria*)  *Methylococcales* (*Gammaproteobacteria*) | −  +  +  −  + | −  +  +  −  − |
| **Nitrification** | *amoA* | *Nitrosophaeria* (*Thaumarchaeota*)  *Hyphomicrobiales* (*Alphaproteobacteria*) | +  − | +  − |
| **Complete denitrification** | *narG*  *nirK* or *nirS*  *norB*  *nosZ* | *Magnetospirilium* (*Alphaproteobacteria*, *Rhodospirilales*)  *Methylocystis* (*Alphaproteobacteria*, *Hyphomicrobiales*)  *Ralstonia* (*Betaproteobacteria*, *Burkholderiales*)  *Burkholderia* (*Betaproteobacteria*, *Burkholderiales*)  *Paraburkholderia* (*Betaproteobacteria*, *Burkholderiales*)  *Dyella* (Gammaproteobacteria, Xanthomonadales)  *Terriglobia* (*Acidobacteria*) | +  −  +  +  +  +  + | +  −  +  +  +  +  + |
| **Dissimilatory Nitrate/Nitrite Reduction to Ammonia (DNRA)** | *nrfA* | *Terriglobia* (*Acidobacteria*) | + | + |

*^#^Phylum/Class/Order/Family/Genus; Upregulated represented by “+”; Downregulated represented by “−“.*

# **Table S14** Genes associated with methane production and consumption (gene name and KEGG Orthology)

| **No.** | **Gene ID** | **Gene name** | **KEGG Orthology** |
| --- | --- | --- | --- |
| **1** | ***phnJ*** | alpha-D-ribose 1-methylphosphonate 5-phosphate C-P lyase | K06163 |
| **2** | ***mcrA*** | methyl-coenzyme M reductase alpha subunit | K00399 |
| **3** | ***mcrB*** | methyl-coenzyme M reductase beta subunit | K00401 |
| **4** | ***mcrC*** | methyl-coenzyme M reductase subunit C | K03421 |
| **5** | ***mmoX*** | methane monooxygenase component A alpha chain | K16157 |
| **6** | ***pmoA-amoA*** | methane/ammonia monooxygenase subunit A | K10944 |
| **7** | ***pmoB-amoB*** | methane/ammonia monooxygenase subunit B | K10945 |
| **8** | ***pmoC-amoC*** | methane/ammonia monooxygenase subunit C | K10946 |

# **Table S15** Genes associated with nitrogen transformation leading to nitrous oxide production and consumption (gene name and KEGG Orthology)

| **No.** | **Gene ID** | **Gene name** | **KEGG Orthology** |
| --- | --- | --- | --- |
| **1** | ***narG*** | nitrate reductase/nitrite oxidoreductase, alpha subunit | K00370 |
| **2** | ***nirK*** | nitrite reductase (NO-forming) | K00368 |
| **3** | ***nirS*** | nitrite reductase (NO-forming) / hydroxylamine reductase | K15864 |
| **4** | ***norB*** | nitric oxide reductase subunit B | K04561 |
| **5** | ***nosZ*** | nitrous-oxide reductase | K00376 |
| **6** | ***nirB*** | nitrite reductase (NADH) large subunit | K00362 |
| **7** | ***nrfA*** | nitrite reductase (cytochrome c-552) | K03385 |
| **8** | ***nifH*** | nitrogenase iron protein *NifH* | K02588 |
| **9** | ***nifD*** | nitrogenase molybdenum-iron protein alpha chain | K02586 |
| **10** | ***nifK*** | nitrogenase molybdenum-iron protein beta chain | K02591 |

# **Figure S1**


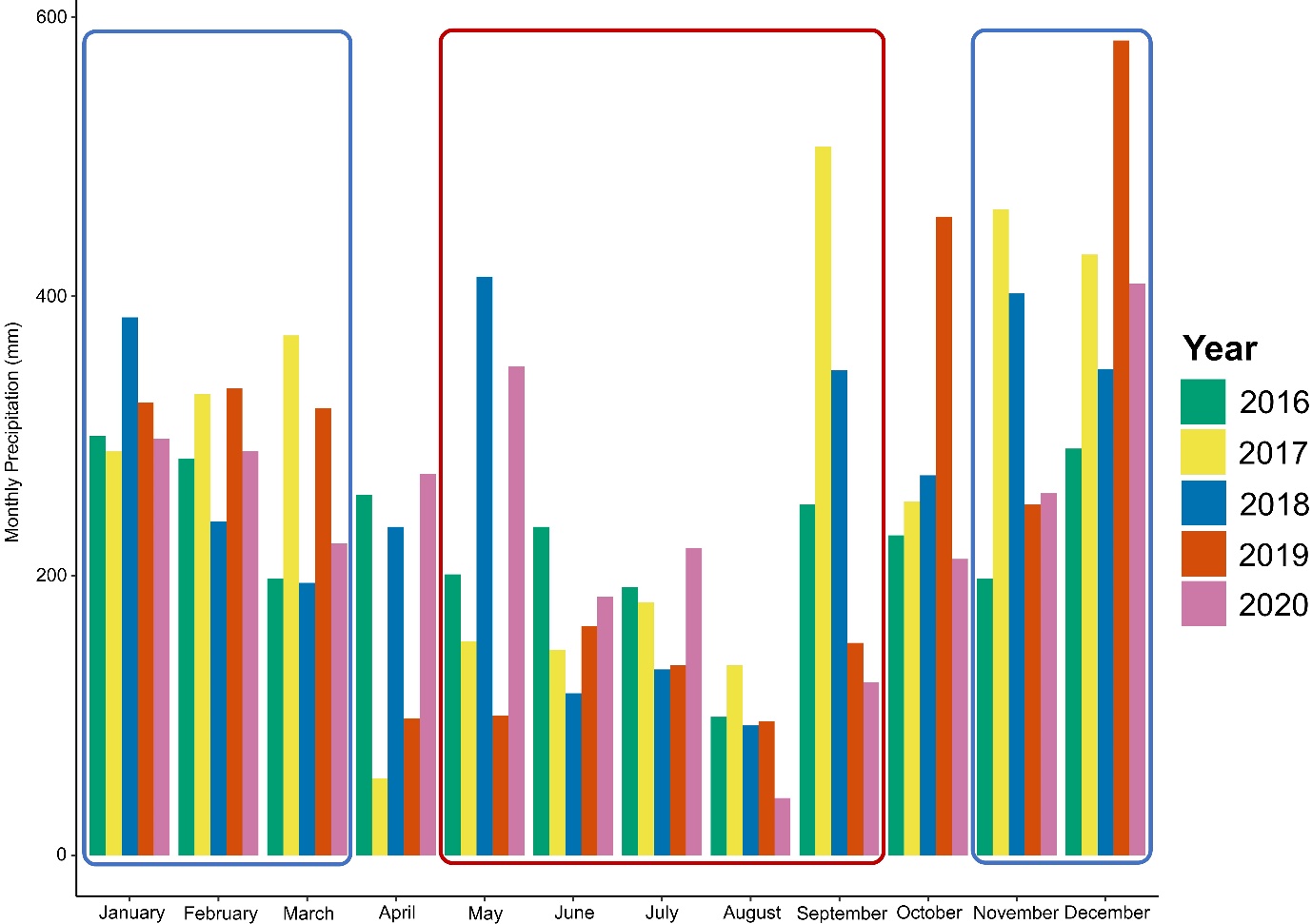


Monthly precipitation from January 2016 to December 2020. The annual precipitation is 2734 mm (2016), 3312 mm (2017), 3175 mm (2018), 3012 mm (2019) and 2880 mm (2020). The wet season (blue outline box), begins in November and ends in March. The dry season (red outline box) is characterized by a drier period from May to September. April and October are transitional months between seasons.

# **Figure S2**


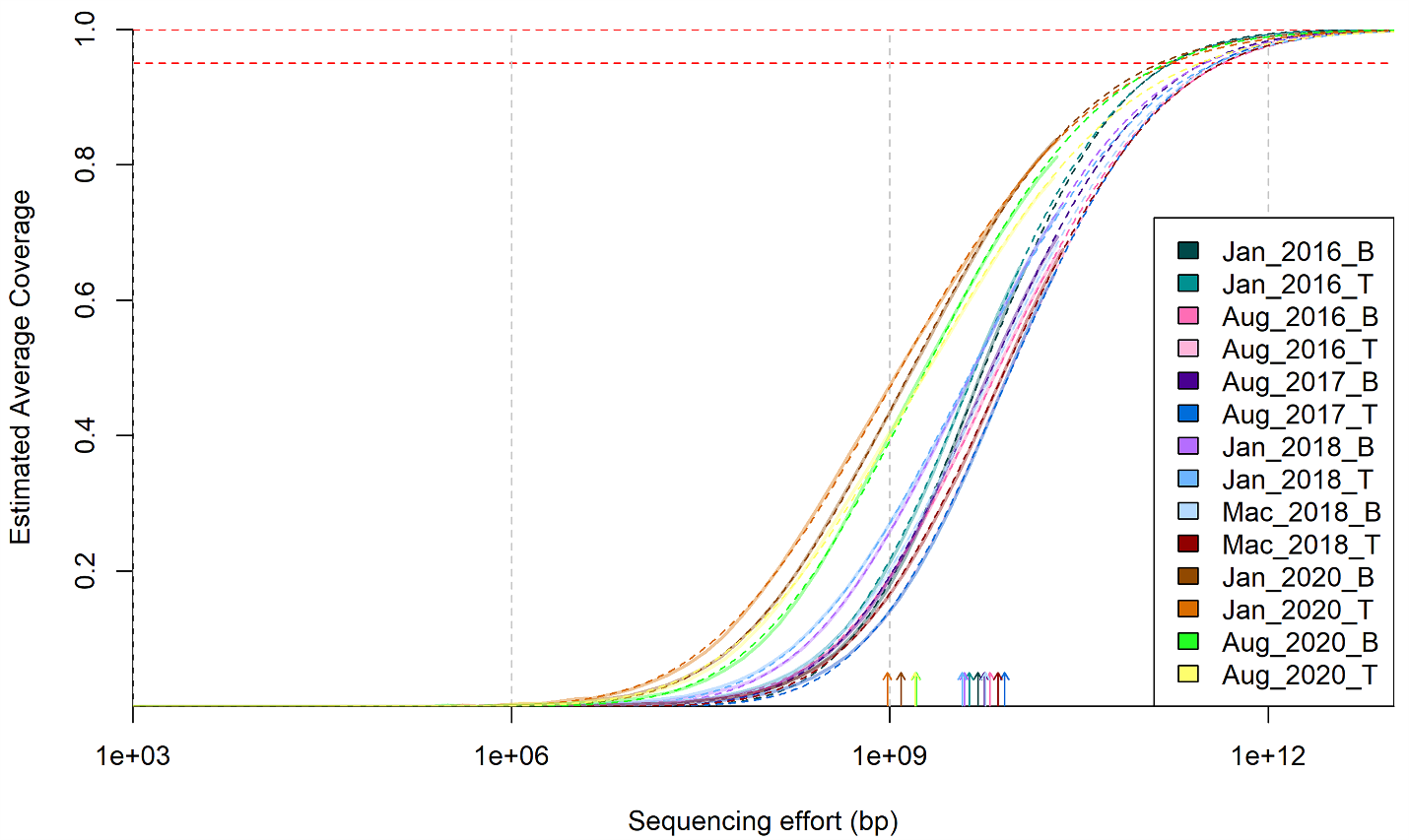


Estimated coverage to actual sequencing effort of tropical peatland samples. Based on predicted values, a sequencing effort of over one tera base pair would be required to achieve near-complete to complete coverage for tropical peat samples. Less sequencing effort is needed to achieve near-complete coverage for oil palm plantation samples, suggesting lesser microbiome diversity than the swamp peat forest.

# **Figure S3**


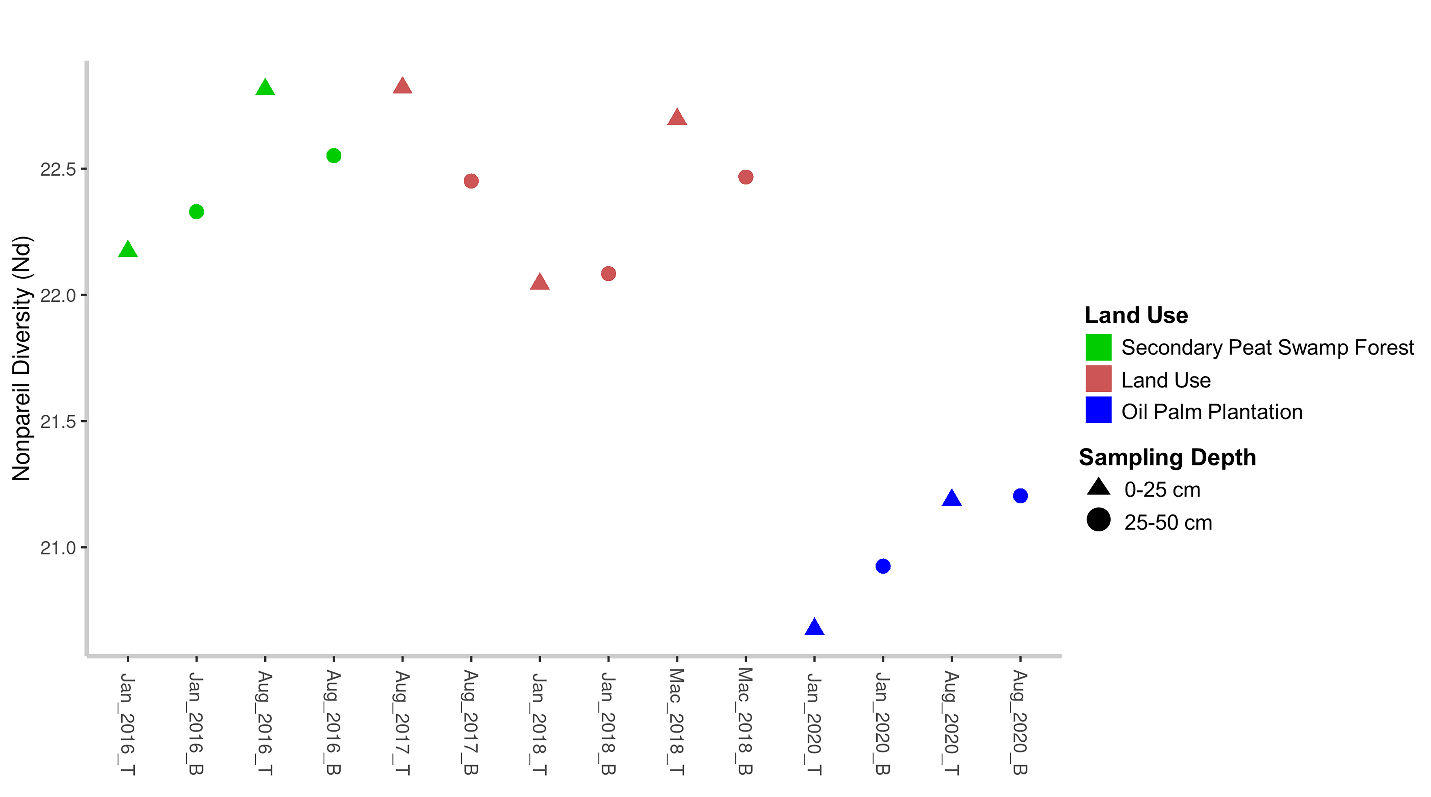


Nonpareil diversity is calculated on forward reads using *kmer* diversity based on reads above 35 base pairs.

# **Figure S4**


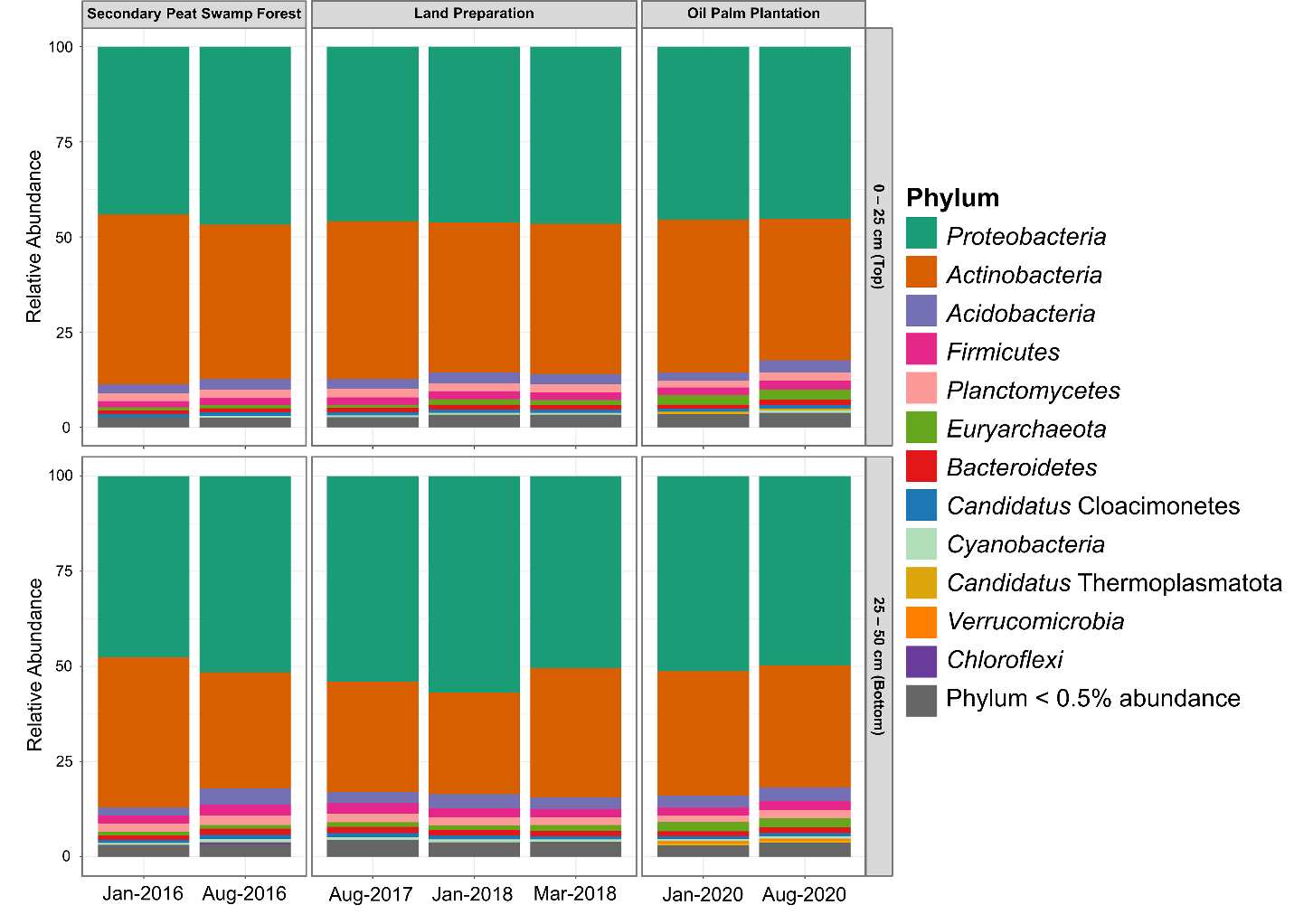


Relative abundance of tropical peatland prokaryotic phyla across temporal land use and sampling depth. Phyla with less than 0.5% relative abundance were grouped.

# **Figure S5**


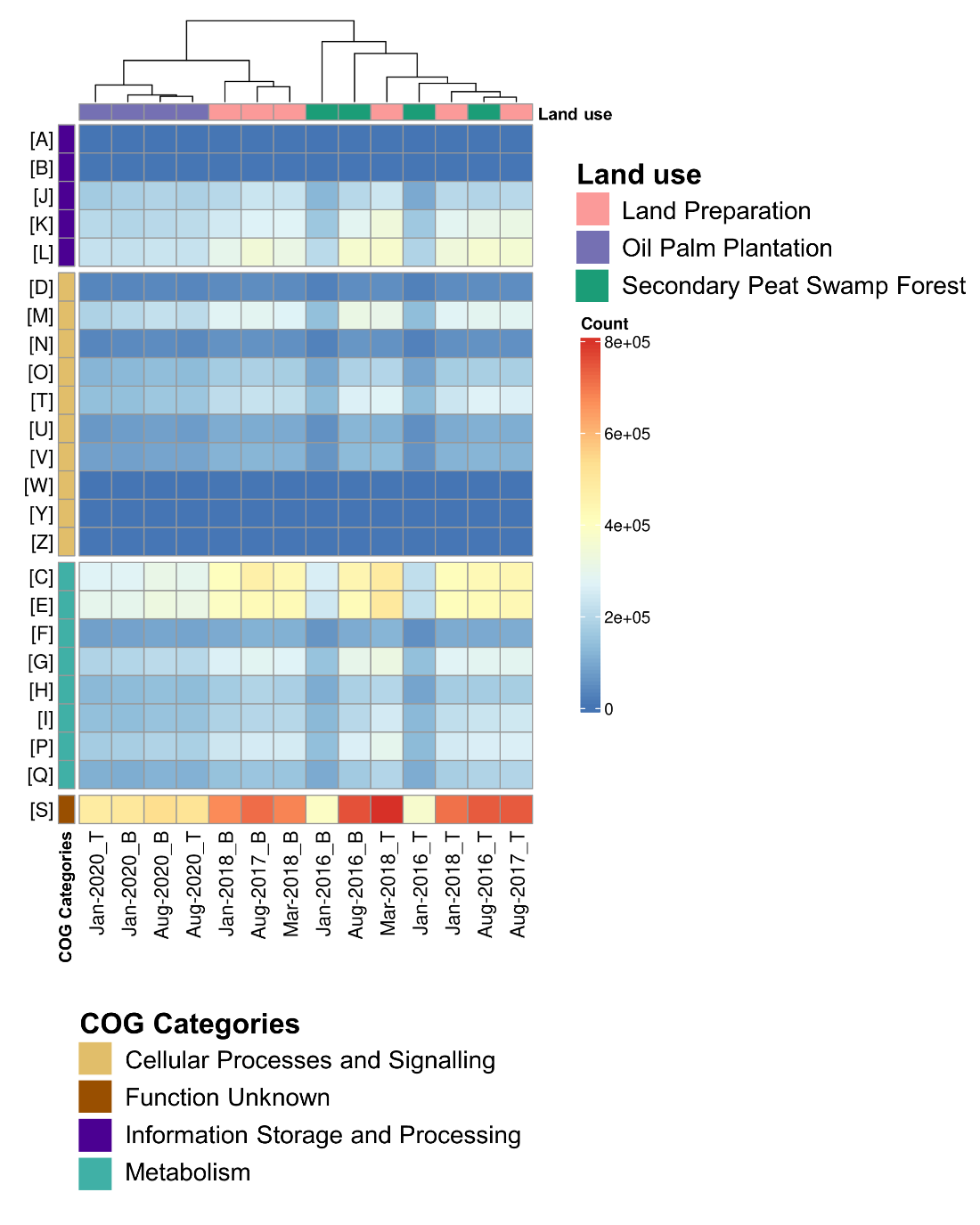


Absolute count for Clusters of Orthologous Genes (COG) categories recovered from tropical peatland samples. The COGs absolute count is from 0 (min) to 806191 (max). Most of the protein-coding sequences were found to be associated with the “Energy Production and Conversion [C]”, “Amino Acid Transport and Metabolism [E]”, “Carbohydrate Transport and Metabolism [G]”, “Translation, Ribosomal Structure and Biogenesis [J]”, “Transcription [K]”, “Replication, Recombination and Repair [L]” and “Cell Wall/Membrane/Envelope Biogenesis”. These COGs were relatively higher in the forest and land preparation samples than in oil palm plantation samples.

# **Figure S6**


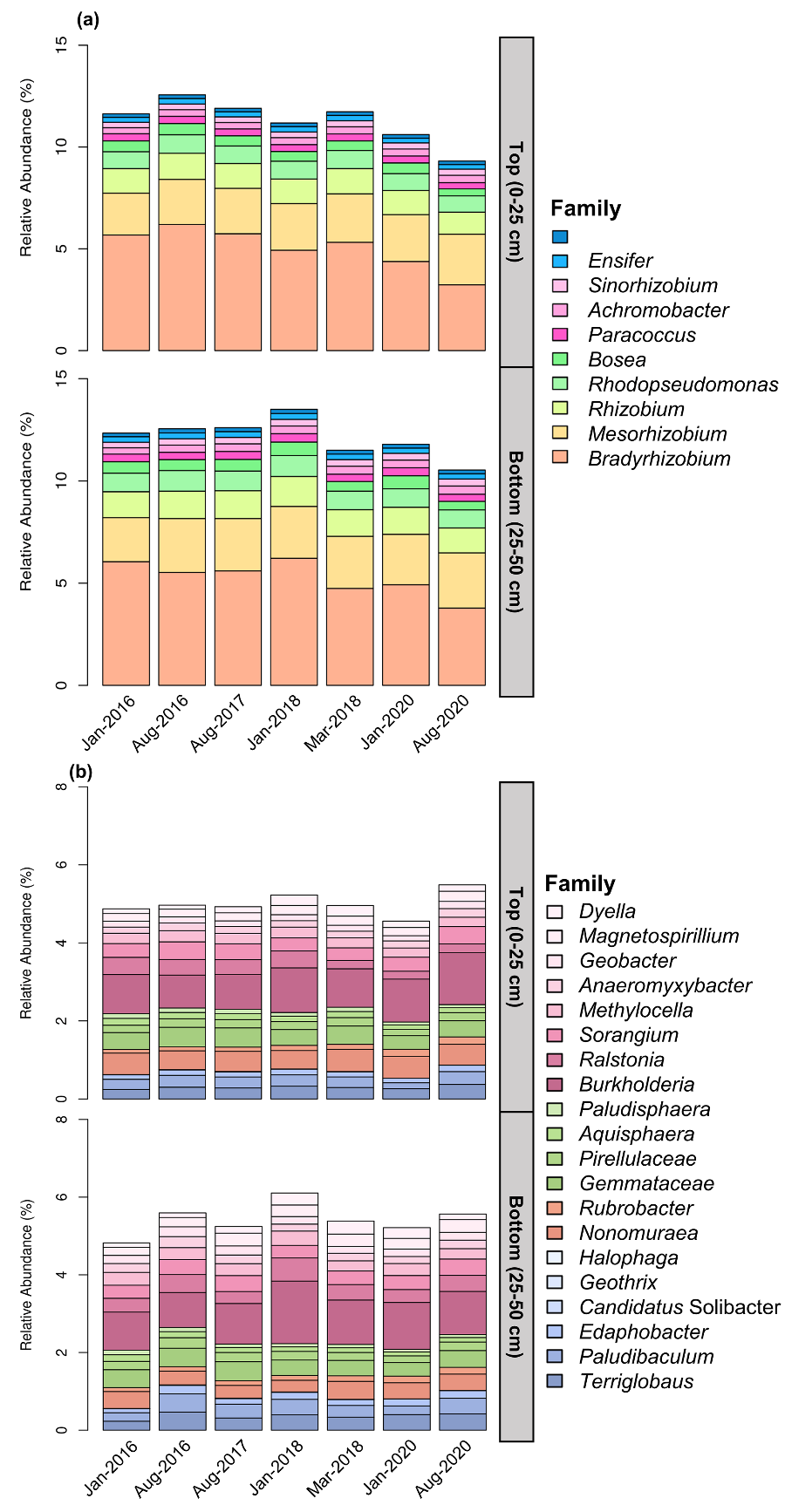


Prokaryotic relative abundance of *nirK*-type denitrifiers (a), *norB*-type denitrifiers (b). Only taxa with relative abundance above 0.1% are shown.

# **Figure S7**


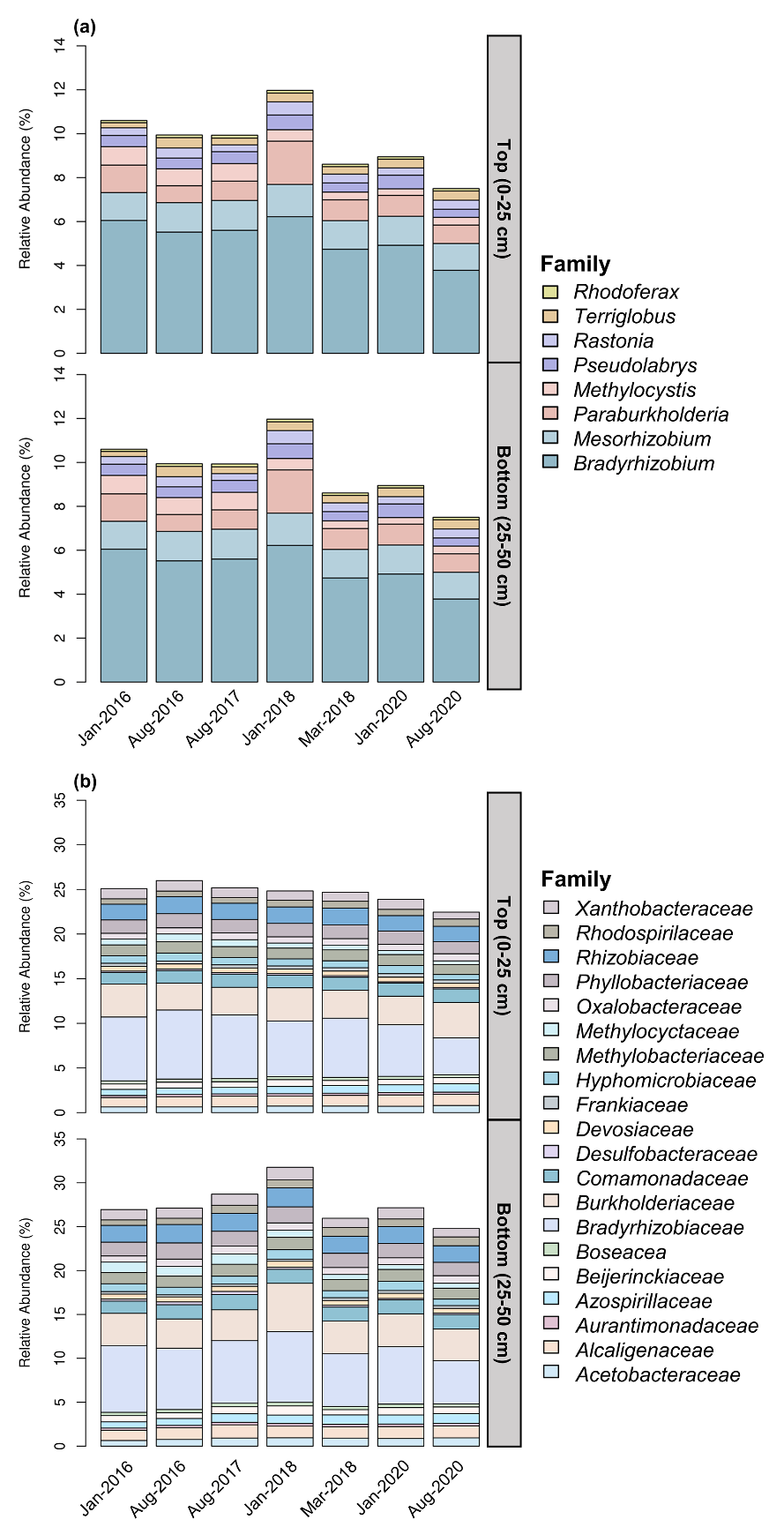


Prokaryotic relative abundance of *nosZ*-type denitrifiers (c) and diazotrophs possessing *nifH* (d). Only taxa with relative abundance above 0.1% are shown.

# **References**

1. Clum A, Huntemann M, Bushnell B, Foster B, Foster B, Roux S, Hajek PP, Varghese N, Mukherjee S, Reddy TBK, Daum C, Yoshinaga Y, O’Malley R, Seshadri R, Kyrpides NC, Eloe-Fadrosh EA, Chen I-MA, Copeland A, Ivanova NN, Segata N. 2021. DOE JGI Metagenome Workflow. mSystems 6:e00804-20.

2. Rodriguez-R LM, Gunturu S, Tiedje JM, Cole JR, Konstantinidis KT, Fodor A. 2018. Nonpareil 3: Fast Estimation of Metagenomic Coverage and Sequence Diversity. mSystems 3:e00039-18.

3. Rodriguez-R LM, Konstantinidis KT. 2014. Estimating coverage in metagenomic data sets and why it matters. The ISME Journal 8:2349-2351.

4. Wood DE, Lu J, Langmead B. 2019. Improved metagenomic analysis with Kraken 2. Genome Biology 20:257.

5. Lu J, Breitwieser FP, Thielen P, Salzberg SL. 2017. Bracken: estimating species abundance in metagenomics data. PeerJ Computer Science 3:e104.

6. Dabdoub SM. 2016. kraken-biom: Enabling interoperative format conversion for Kraken results (Version 1.2).

7. Li D, Liu C-M, Luo R, Sadakane K, Lam T-W. 2015. MEGAHIT: an ultra-fast single-node solution for large and complex metagenomics assembly via succinct de Bruijn graph. Bioinformatics 31:1674-1676.

8. Mikheenko A, Prjibelski A, Saveliev V, Antipov D, Gurevich A. 2018. Versatile genome assembly evaluation with QUAST-LG. Bioinformatics 34:142-150.

9. Mikheenko A, Saveliev V, Gurevich A. 2015. MetaQUAST: evaluation of metagenome assemblies. Bioinformatics 32:1088-1090.

10. Hyatt D, Chen G-L, LoCascio PF, Land ML, Larimer FW, Hauser LJ. 2010. Prodigal: prokaryotic gene recognition and translation initiation site identification. BMC Bioinformatics 11:119.

11. Shen W, Le S, Li Y, Hu F. 2016. SeqKit: A Cross-Platform and Ultrafast Toolkit for FASTA/Q File Manipulation. PLoS ONE 11:e0163962.

12. Cantalapiedra CP, Hernández-Plaza A, Letunic I, Bork P, Huerta-Cepas J. 2021. eggNOG-mapper v2: Functional Annotation, Orthology Assignments, and Domain Prediction at the Metagenomic Scale. Mol Biol Evol 293:5825-5829.

13. Huerta-Cepas J, Szklarczyk D, Heller D, Hernández-Plaza A, Forslund SK, Cook H, Mende DR, Letunic I, Rattei T, Jensen Lars J, von Mering C, Bork P. 2018. eggNOG 5.0: a hierarchical, functionally and phylogenetically annotated orthology resource based on 5090 organisms and 2502 viruses. Nucleic Acids Res 47:D309-D314.

14. Buchfink B, Reuter K, Drost H-G. 2021. Sensitive protein alignments at tree-of-life scale using DIAMOND. Nat Methods 18:366-368.

15. Kang DD, Li F, Kirton E, Thomas A, Egan R, An H, Wang Z. 2019. MetaBAT 2: an adaptive binning algorithm for robust and efficient genome reconstruction from metagenome assemblies. PeerJ 7:e7359-e7359.

16. Alneberg J, Bjarnason BS, de Bruijn I, Schirmer M, Quick J, Ijaz UZ, Lahti L, Loman NJ, Andersson AF, Quince C. 2014. Binning metagenomic contigs by coverage and composition. Nat Methods 11:1144-1146.

17. Wu Y-W, Simmons BA, Singer SW. 2015. MaxBin 2.0: an automated binning algorithm to recover genomes from multiple metagenomic datasets. Bioinformatics 32:605-607.

18. Uritskiy GV, DiRuggiero J, Taylor J. 2018. MetaWRAP—a flexible pipeline for genome-resolved metagenomic data analysis. Microbiome 6:158.

19. Nayfach S, Shi ZJ, Seshadri R, Pollard KS, Kyrpides NC. 2019. New insights from uncultivated genomes of the global human gut microbiome. Nature 568:505-510.

20. Bowers RM, Kyrpides NC, Stepanauskas R, Harmon-Smith M, Doud D, Reddy TBK, Schulz F, Jarett J, Rivers AR, Eloe-Fadrosh EA, Tringe SG, Ivanova NN, Copeland A, Clum A, Becraft ED, Malmstrom RR, Birren B, Podar M, Bork P, Weinstock GM, Garrity GM, Dodsworth JA, Yooseph S, Sutton G, Glöckner FO, Gilbert JA, Nelson WC, Hallam SJ, Jungbluth SP, Ettema TJG, Tighe S, Konstantinidis KT, Liu W-T, Baker BJ, Rattei T, Eisen JA, Hedlund B, McMahon KD, Fierer N, Knight R, Finn R, Cochrane G, Karsch-Mizrachi I, Tyson GW, Rinke C, Kyrpides NC, Schriml L, Garrity GM, Hugenholtz P, Sutton G, et al. 2017. Minimum information about a single amplified genome (MISAG) and a metagenome-assembled genome (MIMAG) of bacteria and archaea. Nature Biotechnology 35:725-731.

21. Shaffer M, Borton MA, McGivern BB, Zayed AA, La Rosa Sabina L, Solden LM, Liu P, Narrowe AB, Rodríguez-Ramos J, Bolduc B, Gazitúa MC, Daly RA, Smith GJ, Vik DR, Pope PB, Sullivan MB, Roux S, Wrighton Kelly C. 2020. DRAM for distilling microbial metabolism to automate the curation of microbiome function. Nucleic Acids Res 48:8883-8900.

22. Parks DH, Chuvochina M, Chaumeil P-A, Rinke C, Mussig AJ, Hugenholtz P. 2020. A complete domain-to-species taxonomy for Bacteria and Archaea. Nature Biotechnology 38:1079-1086.

23. McMurdie PJ, Holmes S. 2013. phyloseq: an R package for reproducible interactive analysis and graphics of microbiome census data. PLoS ONE 8:e61217.

24. Wickham H. 2016. ggplot2: Elegant Graphics for Data Analysis. New York: Springer-Verlag.

25. Oksanen J, Simpson G, Blanchet F, Kindt R, Legendre P, Minchin P, O'Hara R, Solymos P, Stevens M, Szoecs E, Wagner H, Barbour M, Bedward M, Bolker B, Borcard D, Carvalho G, Chirico M, De Caceres M, Durand S, Evangelista H, FitzJohn R, Friendly M, Furneaux B, Hannigan G, Hill M, Lahti L, McGlinn D, Ouellette M, Ribeiro Cunha E, Smith T, Stier A, Ter Braak C, Weedon J. 2022. vegan: Community Ecology Package version 2.6.4.

26. Gu Z, Eils R, Schlesner M. 2016. Complex heatmaps reveal patterns and correlations in multidimensional genomic data. Bioinformatics 32:2847-2849.
